# Satellite DNA-targeted CRISPR–Cas9-mediated editing enables chromosome truncation and elimination in wheat

**DOI:** 10.1016/j.xplc.2026.101833

**Published:** 2026-03-23

**Authors:** Jianyong Chen, Taoran Liu, Yating Xia, Luisa Barth, Jörg Plieske, Heike Gnad, Suriya Tamilselvan-Nattar-Amutha, Zengjun Qi, Stefan Heckmann, Andreas Houben

**Affiliations:** 1 Leibniz Institute of Plant Genetics and Crop Plant Research (IPK), Gatersleben, 06466 Seeland, Germany; 2 State Key Laboratory of Crop Genetics and Germplasm Enhancement & Utilization, Nanjing Agricultural University, Nanjing 210095, China; 3 SGS INSTITUT FRESENIUS GmbH, TraitGenetics Section, Am Schwabeplan 1b, 06466 Seeland OT Gatersleben, Germany; 4 Martin Luther University Halle-Wittenberg, Institute of Agricultural & Nutritional Sciences, Halle, Saale, Germany

Dear Editor,

Although CRISPR–Cas has advanced chromosome engineering in model systems such as *Arabidopsis* (Yasar et al., 2026), manipulation of large-genome crops remains challenging (Supplemental Note 1). Here, we present virus-induced genome editing (VIGE) using satellite repeat-specific single guide RNAs (sgRNAs) as a tool for chromosomal alterations. To minimize lethality caused by genetic loss, we first performed *in planta* chromosome truncation in a wheat–rye B chromosome addition line. The Cas9-expressing line 707 of wheat cv. “Bobwhite” (Wang et al., 2022) was used as a pollinator in a cross with a wheat “Chinese Spring” line carrying the dispensable rye B chromosome (Endo et al., 2008; Chen et al., 2024). B chromosome-positive (B) and Cas9-positive plants were identified using the B-specific satellite D1100 as a fluorescence *in situ* hybridization (FISH) probe and a Cas9-specific primer pair (Supplemental Table 1). Cas9-positive plants carrying a single B were selected to avoid post-editing heterozygosity of the B chromosome. Two sgRNAs were designed (Supplemental Table 1): one targeting the B-specific satellite E3900 and the other targeting the B-specific single-copy gene *DCR400* (Chen et al., 2024) as a control (Figure 1A).

**Figure 1:**
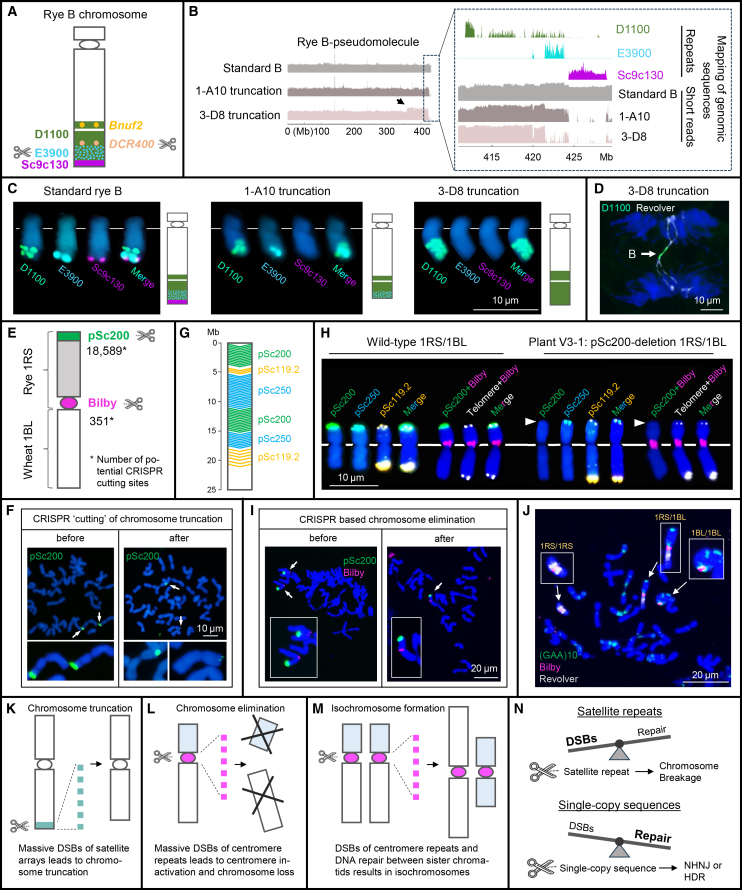
CRISPR–Cas9-mediated truncation and elimination of chromosomes. **(A)** Schematic showing the chromosomal distribution of the B chromosome-specific repeats Sc9c130, E3900, and D1100. *Bnuf2* and *DCR400* are B chromosome-specific single-copy genes. Scissors indicate CRISPR-based cutting targets. **(B)** Comparative short-read sequencing mapping of plants carrying standard B chromosomes and the B truncation lines (1-A10 and 3-D8) against the complete rye B pseudomolecule. Short-read data from plants carrying two standard B chromosomes and from the B truncation lines 1-A10 and 3-D8 were mapped onto the rye B pseudomolecule. An enlarged view shows read mapping and the distribution of D1100 (green), E3900 (blue), and Sc9c130 (violet) repeats in the terminal 400–429 Mb region of the rye B pseudomolecule. **(C)** The standard rye B chromosome and truncated B chromosomes from plants 1-A10 and 3-D8 were analyzed by FISH using the B chromosome-specific repeats D1100 (green), E3900 (blue), and Sc9c130 (violet). Corresponding complete mitotic cells after FISH are shown in Supplemental Figure 3. **(D)** Mitotic anaphase of plant 3-D8 showing a B chromosome bridge. FISH was performed using the B chromosome-specific repeat D1100 (green) and the rye genome-specific repeat Revolver (white). **(E)** Schematic representation of intermingled pSc200, pSc250, and pSc119.2 repeat arrays within the subtelomeric region (0–25 Mb) of the assembled 1RS chromosome arm in the wheat line Zhou8425B (Li et al., 2025). **(F)** CRISPR–Cas9-mediated cleavage of the rye-specific satellite pSc200 (green) resulted in reduced pSc200 FISH signals on one 1RS/1BL chromosome in plant V2-4 (right). The left panel shows the parental wild-type chromosomes after FISH. Arrows indicate typical wild-type pSc200 signals, and the triangle indicates reduced pSc200 FISH signals. **(G)** Schematic representation of intermingled pSc200, pSc250, and pSc119.2 repeat arrays within the subtelomeric region (0–25 Mb) of the assembled 1RS chromosome arm in the wheat line Zhou8425B. **(H)** Chromosome 1RS/1BL of plant V3-1 shows complete loss of pSc200 FISH signals, whereas telomere, pSc250 and pSc119.2 signals are retained. For comparison, wild-type 1RS/1BL chromosomes after FISH are shown using probes specific for pSc200 (green), *Arabidopsis*-type telomere (white), Bilby (magenta), pSc250 (blue), and pSc119.2 (orange). Corresponding complete mitotic cells after FISH are shown in Supplemental Figure 4. White triangles mark deletion of the pSc200 region. **(I)** CRISPR–Cas9-mediated cleavage of the rye centromeric repeat Bilby eliminated one 1RS/1BL chromosome in plant 032 (right). The left panel shows the parental plant of 032 before cutting. FISH was performed using pSc200 (green) and Bilby (magenta). Corresponding interphase nuclei are shown in Supplemental Figure 5. **(J)** Plant 0311 possesses a monosomic 1RS/1BL, a monosomic 1RS/1RS isochromosome, and a monosomic 1BL/1BL isochromosome. FISH was performed using the centromeric probes (GAA)_10_ (green) and Bilby (magenta), as well as the rye genome-specific probe Revolver (white). In all FISH images, chromosomes are counterstained with DAPI (blue). **(K–N)** Summary of outcomes of CRISPR–Cas9-induced cleavage of satellite DNA. **(K)** CRISPR–Cas9-induced cleavage of satellite DNA leads to chromosome truncation. DSBs, double-strand breaks. **(L)** CRISPR–Cas9-induced cleavage of a CENH3-associated centromeric repeat results in centromere loss and chromosome elimination. **(M)** Alternatively, DNA DSBs in centromeric repeats, followed by erroneous DNA repair between sister chromatids, result in monocentric isochromosomes. **(N)** Outcomes differ when Cas9 targets satellite repeats vs. single-copy sequences. When CRISPR targets a satellite repeat, it generates numerous DSBs, overwhelming the cell’s DNA repair capacity and leading to chromosome breakage. In contrast, targeting a single-copy sequence produces a single DSB that can be efficiently repaired via non-homologous end joining (NHEJ) or homology-directed repair (HDR).

Both gRNAs were delivered via Barley stripe mosaic virus (BSMV) (Tamilselvan-Nattar-Amutha et al., 2023) to five Cas9- and B-positive F_1_ plants each. This generation was designated M_0_, as genome editing is initiated upon the combination of sgRNA and Cas9. Only the resulting virus-free M_1_ plants were subsequently analyzed, as the absence of the virus prevents further editing activity. The M_1_ plants were genotyped using a primer pair specific for the B-specific gene *Bnuf2* (Figure 1A and Supplemental Table 1). A primer pair specific for the subtelomeric B-specific repeat Sc9c130 (Supplemental Table 1) was used to identify truncations of the long arm of the B chromosome (Figure 1A), as CRISPR-induced breaks within the E3900 array may delete the region distal to the break. In the experiment targeting repeat Sc9c130, 82 of 179 PCR-genotyped plants contained a B chromosome, of which eight (9.8%) were negative for Sc9c130 (Supplemental Figure 1). In contrast, in the experiment targeting the single-copy gene *DCR400,* none of the 86 B-containing plants showed a terminal deletion (Supplemental Table 2).

To validate the loss of the Sc9c130 repeat, we randomly selected two RT–PCR-confirmed, virus-free plants, 1-A10 and 3-D8, carrying a Sc9c130-free B chromosome for whole-genome short-read sequencing (WGS) at approximately 2-fold genome coverage. The reads were aligned to the rye B reference sequence, together with WGS data from wheat plants carrying two standard B chromosomes as a control (Figure 1B). Significantly reduced read mapping was observed in 1-A10 and 3-D8 at the distal 424–429 Mb region, corresponding to the Sc9c130 region of the B chromosome, confirming a terminal deletion. Additionally, 3-D8 showed increased read coverage in the 380–421 Mb region of the B chromosome, suggesting a segmental duplication (Figure 1B and Supplemental Figure 2). Zooming into the terminal region revealed that plant 1-A10 retained most E3900 repeat copies, whereas 3-D8 showed a substantial loss of E3900.

FISH analysis of 1-A10 and 3-D8, together with plants carrying an intact B chromosome, using the B-specific probes D1100, E3900, and Sc9c130, corroborated these findings (Figure 1C and Supplemental Figure 3). In the standard B chromosome, D1100 occupies nearly the entire terminal region of the long arm, except for a small segment. E3900 partially overlaps with D1100 near the subtelomere, whereas Sc9c130 is located at the chromosome end (Figures 1A and 1C). CRISPR–Cas9-mediated cleavage of E3900 in 1-A10 and 3-D8 resulted in the loss of the subtelomeric Sc9c130 FISH signal (Figure 1C and Supplemental Figure 3). Interestingly, 3-D8 showed an enhanced D1100 signal compared with the standard B chromosome (Figure 1C and Supplemental Figure 3), indicating a duplication involving the D1100 array. This pattern may arise from extension of a distal break end over a proximal break end before ligation, followed by deletion at a more distal breakpoint. Alternatively, a breakage–fusion–bridge cycle, which involves the fusion of proximal break ends between sister chromatids followed by random mitotic breakage of the resulting dicentric chromosome in several subsequent mitotic cycles, could be responsible for the observed segmental duplication in the truncated chromosome. This is supported by the occasional observation of a mitotic anaphase bridge involving the B chromosome in plant 3-D8 (Figure 1D). Thus, CRISPR–Cas9-mediated cleavage of rye B-specific satellite repeats enables downsizing of B chromosomes.

To test whether the same approach could be used to reduce the copy number of a standard chromosomal satellite repeat, we targeted the rye–wheat Robertsonian translocation chromosome 1RS/1BL. The Cas9-expressing wheat line (Wang et al., 2022) carries this translocation chromosome, with the rye-specific subtelomeric pSc200 satellite located on its short arm (Figure 1E). A pSc200-specific sgRNA was introduced into five Cas9-positive plants via BSMV (Supplemental Table 1). Seventeen of 18 plants exhibited a substantial reduction in pSc200 FISH signals (Supplemental Tables 3 and 4 and Figure 1F). Nine of 18 plants showed loss of pSc200 signals on at least one 1RS/1BL chromosome, and three plants (17%) displayed complete deletion of pSc200 on both 1RS/1BL chromosomes (Supplemental Tables 3 and 4). In the assembled genome of the 1RS/1BL-carrying wheat line Zhou8425B (Li et al., 2025), we identified 18 589 target sites with 100% identity to the pSc200 sgRNA within a 15 Mb subtelomeric region (Figure 1E and Supplemental Table 6).

The 1RS arm features a distal pSc200 array, followed by sequential pSc119.2, pSc250, pSc200, and pSc250 arrays, with a final pSc119.2 array located toward the centromere (Figure 1G). To determine whether CRISPR-mediated deletion of pSc200 also deleted the adjacent pSc250 and pSc119.2 arrays, we performed FISH using pSc250- and pSc119.2-specific probes on plant V3-1, which is homozygous for the pSc200 deletion, as well as a wild-type control (Figure 1H). Despite the absence of pSc200 in V3-1, both pSc250 and pSc119.2 repeats remained at the distal end of chromosome 1RS (Figure 1H and Supplemental Figure 4A), indicating that adjacent repeat arrays toward the centromere were unaffected. Compared with the parental wild-type plant, which shows two adjacent pSc119.2 signal clusters in the subtelomeric region of 1RS, the pSc200-deletion plant retained only one (Figures 1H and Supplemental Figure 4A).

To assess whether the induced chromosome truncations were sealed by *de novo* telomere addition, we analyzed the V3-1 M_1 plant_, which exhibited complete deletion of pSc200 signals on both 1RS arms. Telomere-FISH revealed terminal signals on 1RS. Although the wild-type 1RS/1BL chromosome showed stronger telomere signals, the broken chromosome ends of 1RS were sealed with telomeres (Figure 1H and Supplemental Figure 4B).

Next, we tested whether this approach could be used to eliminate an entire chromosome. We focused on the 1RS/1BL chromosome, which carries the rye-specific centromeric repeat *Bilby* that interacts with centromeric histone H3 (CENH3), thereby indicating that the rye-derived centromere of 1RS/1BL is active (Houben et al., 2011; Karimi-Ashtiyani et al., 2021). An sgRNA (Supplemental Table 1) recognizing 351 target sites with 100% identity within the Bilby array (Figure 1E and Supplemental Table 6) was delivered via BSMV into five plants. To detect loss of 1RS/1BL, 16 M_1_ plants were analyzed by FISH using the rye probes pSc200 and Bilby. Among these, four (25%) plants were identified as monosomic for 1RS/1BL, indicating loss of one copy of the chromosome (Figure 1I and Supplemental Tables 3 and 5). This result demonstrates that targeted chromosome elimination can be achieved through CRISPR–Cas9-mediated cleavage of centromeric DNA. In addition, plant 0311 carried monosomic 1RS/1BL, as well as 1RS/1RS and 1BL/1BL chromosomes, suggesting that induced double-strand breaks followed by erroneous DNA repair between sister chromatids can generate short- and long-arm monocentric isochromosomes (Figure 1J). In this case, cleavage of centromeric repeats increased the number of chromosomes. To evaluate the specificity of this approach, we performed FISH karyotyping on two selfed progenies derived from wheat monosomic 1RS/1BL (2*n* = 41) plants 032 and 034 (Supplemental Table 5). Both analysed plants contained all wheat chromosomes (2*n* = 41) except one copy of 1RS/1BL (Supplemental Figure 6).

To investigate whether the same strategy could be used to eliminatenative wheat chromosomes, we targeted the wheat centromeric satellite CentT566 (Su et al., 2019). *In silico* analysis predicted that the CentT566-gRNA selectively targets only four chromosomes—1RS/1BL, 5B, 6B, and 7B—with over 20 perfect matches each (Supplemental Table 6). The centromeric 220–225 Mb region on chromosome 5B harbored the highest density of targets (218 sites; Supplemental Figure 7A and Supplemental Table 6), whereas the centromeres of 1RS/1BL, 6B, and 7B each contained approximately 80–98 target sites (Supplemental Figure 7A and Supplemental Table 6).

CentT566-gRNA was delivered into five plants of the Cas9-positive wheat line via VIGE. Thirty M_1_ plants were analyzed by FISH karyotyping to detect chromosome rearrangements. M_1_ plant 2-20 exhibited a monosomic chromosome 5B, a monosomic 5BL/5BL isochromosome, and a monosomic telocentric 5BS chromosome (Supplemental Figure 7B). To further evaluate this phenomenon, we karyotyped 17 metaphase cells from plant 2-20. Among these, only six retained 5BS, whereas 14 contained the 5BL/5BL isochromosome and 15 carried chromosome 5B. The lower frequency of 5BS cannot be explained by loss during chromosome preparation; instead, mitotic instability is the likely cause. The presence of a 5BL/5BL isochromosome and a telocentric 5BS strongly suggests that CRISPR–Cas9 induced chromosome rearrangements at the centromere of chromosome 5B.

In conclusion, VIGE-mediated CRISPR targeting of satellite repeats expands the chromosome engineering toolkit. The high efficiency likely arises from numerous double-strand breaks that overwhelm the repair machinery, leading to truncation, loss, or isochromosome formation (Figures 1K–1N). Consistent with this interpretation, a very low frequency of chromosomal changes has been reported when using a single-copy gRNA target (Samach et al., 2023). Although limited by lower specificity and repeat-dependent cleavage sites, satellite-specific gRNAs enable targeted chromosome truncation and elimination. This strategy facilitates the transfer of chromosomal segments from wild relatives into wheat, enhances genetic diversity and breeding potential, and enables the generation of minichromosomes as future engineered genetic platforms (Supplemental Note 2).

## Data and code availability

The short-read sequencing data for plant 1-A10 (run accession number: ERR14952793) and 3-D8 (run accession number: ERR14952833) are available in the European Nucleotide Archive (ENA) under project PRJEB89395. Data for wheat with standard B chromosomes (Chen et al., 2024) are available under accession PRJEB69479 (run accession number: ERR12870557).

## Funding

This work was supported by the 10.13039/501100001659German Research Foundation (projects 559365585 and 395162636 to A.H.) and the 10.13039/501100004543China Scholarship Council (CSC202006850005 to J.C.).

## Acknowledgments

We gratefully acknowledge T. Endo (Japan) for providing valuable wheat seeds carrying the rye B chromosome and E. Akhunov (USA) for supplying the high-Cas9 wheat line 707. We thank I. Schubert (IPK, Germany) for fruitful discussions. We also thank O. Weiss, K. Kumke, and J. Lorenz for excellent technical assistance; A. Fiebig and M. Maruschewski (IPK, Germany) for support with sequence submission; and J. Fuchs for assistance with figure preparation. No conflict of interest declared.

## Author contributions

J.C. performed most experiments, including plant cultivation, FISH, VIGE, PCR, and data analysis. T.L., L.B., S.T.-N.-A., and S.H. performed VIGE. Y.X. and Z.Q. performed karyotyping. J.P. and H.G. conducted genotyping. A.H. supervised the research project. J.C. and A.H. wrote the manuscript with input from all coauthors.

## Supplemental information

Document S1. Supplemental Tables S1–S5, Supplemental Notes S1 and S2, and Supplemental Methods

Document S2. Supplemental Figures S1–S8

Supplemental Table 6. Genome-wide prediction of target sites for sgRNAs used in this study

Document S3. Article plus supplemental information
