# Supplementary material for: Satellite DNA-targeted CRISPR–Cas9-mediated editing enables chromosome truncation and elimination in wheat
Source: Plant Commun. 2026 Mar 23;7(7):101833. doi: 10.1016/j.xplc.2026.101833 (PMC13370215; doi:10.1016/j.xplc.2026.101833)
Supplement: Document S1. Supplemental Tables S1–S5, Supplemental Notes S1 and S2, and Supplemental Methods [file mmc1.pdf]

Plant Communications, Volume 7

Supplemental information

Satellite DNA-targeted CRISPR–Cas9-mediated editing enables chromosome truncation and elimination in wheat

Jianyong Chen, Taoran Liu, Yating Xia, Luisa Barth, Jörg Plieske, Heike Gnad, Suriya Tamilselvan-Nattar-Amutha, Zengjun Qi, Stefan Heckmann, and Andreas Houben

Supplementary information

Satellite DNA-targeted CRISPR/Cas9-mediated editing enables chromosome truncation and elimination in wheat

Jianyong Chen[1*], Taoran Liu[1], Yating Xia[2], Luisa Barth[1], Jörg Plieske[3], Heike Gnad[3], Suriya Tamilselvan-Nattar-Amutha[1], Zengjun Qi[2], Stefan Heckmann[1] & Andreas Houben[1,4*]

[1] Leibniz Institute of Plant Genetics and Crop Plant Research (IPK), Gatersleben, 06466 Seeland, Germany

[2] State Key Laboratory of Crop Genetics and Germplasm Enhancement & Utilization, Nanjing Agricultural University, 210095 Nanjing, China

[3] SGS INSTITUT FRESENIUS GmbH, TraitGenetics Section, Am Schwabeplan 1b, 06466 Seeland OT Gatersleben, Germany

[4] Martin Luther University Halle Wittenberg, Institute of Agricultural & Nutritional Sciences, Halle, Saale, Germany

* Corresponding Authors:

Andreas Houben, e-mail: houben@ipk-gatersleben.de

Jianyong Chen, e-mail: chenj@ipk-gatersleben.de

Supplementary Note 1

Plant chromosome engineering, aimed at enhancing genetic variation, began with the pioneering work of (Sears 1956), who used X-ray irradiation to induce chromosome translocations between wheat and its wild relative, *Aegilops umbellulata.* Other methods to change the combination of genes or structure of plant chromosomes included

heterozygous combination of already rearranged chromosome complements (Schubert and Rieger 1985; Schubert and Oud 1997), the application of the gametocidal system (Shi and Endo 1997), the breakage-fusion-bridge cycle (Lukaszewski 1997) or the Pairing Homoeologous (Ph) system (Riley and Chapman 1958; Sears and Okamoto 1958).

With the development of CRISPR/Cas9 systems, the targeted depletion of chromosomes in mammalian cell cultures has become feasible through the application of single or multiplexed guide RNAs (Zuo et al. 2017; Adikusuma et al. 2017; Hashizume et al. 2025). However, the elimination of an entire plant chromosome by this technology has not yet been demonstrated. So far, CRISPR/Cas9 targeting centromeric and rDNA tandem repeats has been used to induce cell death in specific organs and at particular time points in a controlled manner in *A. thaliana* (Schindele et al. 2022; Gehrke et al. 2023). Chromosome rearrangements using guide RNA (gRNA) targeting a unique genomic sequence are now possible in plants, as demonstrated in *Arabidopsis thaliana* (Schmidt et al. 2020; Schmidt et al. 2019; Ronspies et al. 2025), maize (Schwartz et al. 2020), and wheat (Zhao et al. 2025).

Notable, terminal chromosome truncation, chromosome loss and segmental duplications as well as evidence of bridges that could explain some of the observed phenomena following DSB induction. This was obtained at low frequency but using a single copy gRNA target, suggesting that defective repair is not exclusively taking place when repetitive arrays are targeted (Samach et al. 2023).

Another goal in plant chromosome engineering is the generation of minichromosomes, which could be used to stack genes of interest from any source, potentially serving as the first step toward synthesizing a complete engineered plant genome. A 'top-down' approach is preferable to *de novo* minichromosome synthesis, as it begins with the native chromosomes and shortens chromosome arms as much as possible to minimize dosage effects and to achieve phenotypic neutrality (reviewed by (Birchler et al. 2024)). A pioneering study used the *Agrobacterium*-mediated insertion of the telomere repeat to

induce chromosomal truncation in maize (Yu et al. 2006). Later, this method was applied to *Arabidopsis* (Nelson et al. 2011; Teo et al. 2011), barley (Kapusi et al. 2012), rice (Xu et al. 2012), wheat (Yuan et al. 2017), and *Brassica* (Yan et al. 2017). The drawback of the hitherto performed plant chromosome-truncation experiments is the largely random downsizing of chromosomes. For a recent review of plant genome editing see (Puchta and Houben 2024).

Supplementary Note 2

Although heterochromatic features typically lower the CRISPR/Cas9 mutagenesis efficiency for single-copy sequences, as observed across multiple species, including plants (Weiss et al. 2022; Daer et al. 2017), centromeric and noncentromeric satellite DNA arrays proved suitable for CRISPR/Cas9-mediated chromosome engineering. This success aligns with the established use of Cas9 for the *in situ* detection of various satellite repeats, which has been demonstrated in living (Dreissig et al. 2017; Anton et al. 2014), as well as in fixed cells, across species (Ishii et al. 2019; Potlapalli et al. 2025; Potlapalli et al. 2024; Potlapalli et al. 2020; Deng et al. 2015). Thus, satellite repeats are effective targets for CRISPR/Cas-based chromosome engineering, despite their potentially less favourable heterochromatic chromatin features. Most likely, the high copy number compensates for local inhibition of CRISPR/Cas9-based DNA fragmentation.

Unlike CRISPR-induced cutting of 45S rDNA and 178-bp centromere repeats in diploid *A. thaliana*, which triggers cell death (Schindele et al. 2022; Gehrke et al. 2023), polyploid wheat and B chromosomes tolerate extensive DNA loss. This combination provides a powerful platform for developing and testing chromosome-engineering approaches in complex plant genomes. This strategy can be applied even to generate minichromosomes via a ‘top-down’ approach. Unlike the *Agrobacterium*-mediated telomere insertion for achieving random chromosome truncation (Yu et al. 2007; Yu et al. 2006), our approach enables the precise targeting of specific chromosomes by cleaving defined

pericentromeric or other satellite repeats, thereby facilitating the engineering of size-reduced chromosomes as a future shuttle system.

Furthermore, targeted chromosome elimination can accelerate breeding efforts in polyploid crops. For example, a wheat-rye 6R addition line could be crossed with a 6D nullisomic wheat line to produce a double monosomic 6R/6D line, allowing the wheat and rye homeologs to pair and recombine during meiosis in the *ph* mutant background. However, complete wheat hypoploid stocks (nullisomics, monosomics) are limited to the genotype ‘Chinese Spring’ (Endo and Gill 1996; Sears 1954). Therefore, developing a CRISPR-based targeted chromosome elimination in diverse wheat genotypes could facilitate subsequent alien chromosome introgression using the *Ph* system. Whether the elimination of an entire paternal chromosome set by CRISPR-mediated centromere-repeat targeting can effectively serve as a method for generating haploids remains to be tested. For this aim, the centromere repeat composition must be either distinct between the haploid inducer expressing the gRNA/CRISPR construct and the target genotype, or the CRISPR-triggered chromosome elimination must be restricted to the male or female gametes of the haploid inducer.

The VIGE system represents a highly efficient platform for chromosome engineering in crops. Once a Cas9 transgenic plant line has been established, different repeats can be readily targeted by modifying the BSMV vectors carrying the corresponding sgRNAs through simple cloning steps. This strategy enables rapid, flexible genome editing by bypassing the lengthy transformation steps typically required, as shown in our study. In the future, more efficient virus-based approaches can further improve the efficiency of the CRISPR/Cas system. The application of an intronized Cas9 gene might be one way, because the incorporation of introns into the coding sequence strongly enhanced the editing performance in dicots (Grützner et al, 2021) and monocots (Lawrenson et al, 2024). or complemented to enable controlled editing of single-copy loci. Application of the highly efficient Cas enzyme might enable controlled editing of single-copy loci and target repeats for simultaneous chromosome and gene engineering in the future.

Materials and methods

Plant material

Wheat (*Triticum aestivum* L., cv. ‘Chinese Spring’) with two standard rye B chromosomes (Endo et al. 2008) and transgenic wheat cv. ‘Bobwhite’ with high levels of Cas9 expression (high-Cas9 line 707) (Wang et al. 2022) were grown in glasshouses (Gatersleben, Germany). Wheat cv. ‘Chinese Spring’ with two rye B chromosomes was pollinated by the high-Cas9 line 707 to create Cas9-expressing wheat with rye B chromosomes. Cas9-positive plants were selected by PCR using the primer pair zCas9-F and zCas9seq1(Supplementary Table 1) (Wang et al. 2022).

Genomic DNA isolation

Leaf segments (1 - 3 cm) from rye and wheat were frozen in tubes by immersion in liquid nitrogen and pulverized by bead beating in a vibrating mill (MM400, Retsch, frequency: 30/s, 1 min). Extraction buffer (1.21 g Tris, 4.09 g NaCl, 1.86 g EDTA, diluted in 100 ml H_20; 1.2 ml/sample) was added to each tube and incubated for 15 min at 65 °C. Tubes were allowed to cool for 1 min at room temperature (RT). Chloroform: isoamyl alcohol (24:1, 600 µl) was added, and the solution was manually agitated. The homogenate debris was pelleted by centrifugation (13,000 g for 2 min), and the supernatant containing DNA was transferred to a fresh tube. The DNA was precipitated in 700 µl isopropanol, pelleted, and washed twice in 70% ethanol by repeated vortexing and centrifugation (13,000 g for 2 min). The pellet was air-dried until all ethanol was evaporated, and the DNA was dissolved in 50 µl of dH20. The DNA concentration was determined by spectrophotometry (Thermo Scientific NanoDrop One) and the Qubit device (DNA HS assay kit, Thermo Fisher Scientific Inc, Waltham, MA, USA).

Preparation of chromosome spreads

To prepare mitotic chromosomes, seeds were germinated on wet filter paper at room temperature (RT) for 2-3 days. Excised roots were treated with ice-cold water for 24 h for

cell cycle synchronization, fixed in 3:1 ethanol: acetic acid for 1-3 days at RT, and stored in 70% ethanol at -20 °C. To prepare meiotic chromosomes, young spikes were collected when the 1/3 of the flag leaves were out and fixed in 3:1 ethanol: acetic acid for 3 days at RT. Roots or young anthers were treated with 45% acetic acid between 10 and 120 min before being transferred onto a glass slide. Meristematic cells were then isolated in a droplet of 45% acetic acid under a coverslip. After 2 sec of heating over an ethanol burner, the meristem was squashed between the slide and coverslip. Finally, the coverslip was removed after freezing it in liquid nitrogen, and the slide was kept in 99.8% ethanol at -20 °C until use.

Fluorescence in situ hybridization (FISH), probe generation and microscopy

The PCR products and plasmids used as FISH probes were fluorescence-labelled by nick translation (NT Labeling Kit, Jena Bioscience). The repeat Sc9c130 was obtained by PCR. The following plamids were used: pTZE3900, containing a 3.9 kb fragment of the repeat E3900 (Blunden et al. 1993), pUC119-Revolver, containing a 89 bp sequence of the rye-genome specific repeat Revolver (Tomita et al. 2008), pUC18-pSc200 containing a 521 bp fragment of the repeat pSc200 (Vershinin et al. 1995), and a 3.4 kb fragment of the rye-specific centromeric repeat Bilby (Francki 2001). The following oligonucleotide (oligo) probes, each labeled at the 5′ end with a fluorophore, were used in this study: four oligos (30 nt - 40 nt, Oligo-D1100-mix) with fluorescein isothiocyanate (FITC) targeted the sequence of the repeat D1100 (Sandery et al. 1990a, b); Oligo-pSc200-6 targeting the pSc200 repeat (Guo et al. 2019), labeled with 6-carboxyfluorescein (FAM); Oligo-pSc250 targeting the pSc250 repeat (Fu et al. 2015), labeled with 5(6)-carboxytetramethylrhodamine (TAMRA); Oligo-pSc119.2-1 targeting the pSc119.2 repeat and microsatellite $(GAA)_{10}$ (Huang et al. 2018), labeled with FAM; and an oligo probe targeting the *Arabidopsis*-type telomere sequence, also labeled with TAMRA. The sequences of primers and oligo probes are listed in Supplementary Table 1.

For FISH, chromosomes were denatured in a NaOH - 70% ethanol solution (6 mg/ml) for 5 min at RT, then washed, dehydrated in a series of increasing ethanol concentrations

(70%, 90%, and 99.8%) for 5 min each, and air-dried. For each FISH probe, 1 µl of probe (10 mM for oligo probes;50-75 ng/µl for probes generated by nick translation) was added to 10 µl hybridization mixture (Aliyeva-Schnorr et al. 2015), denatured at 99 °C for 10 min and stored immediately at -20 °C until use. The mixture was added to the air-dried slides, sealed with coverslips, and incubated in a moist chamber at 37 °C for 12-24 h. After hybridization, slides were washed in 2x SSC for 20 min at 58 °C and distilled water at RT for 2 min, and air dried. Finally, 8 µl 4',6-diamidino-2-phenylindole (DAPI) solution (1 µg/mL, DAPI/antifade solution) in antifade was added to each slide and sealed with a coverslip. Microscope images were taken using an Olympus BX61 fluorescence microscope equipped with an ORCA-ER CCD camera (Hamamatsu). Images were analyzed using the cellSens Dimension software (Olympus, v1.11) and Adobe Photoshop (v13.0).

Karyotyping of wheat chromosomes

For wheat karyotyping, slide preparation followed a modified protocol in which root tips were treated as described by (Huang et al. 2018) to accumulate metaphase chromosomes. Next, root meristems were transferred into a 0.5 mL microcentrifuge tube containing enzymatic digestion solution (for 10 ml solution, 0.3 g cellulase, 0.1 g pectinases, 2 ml 5× citrate buffer (pH 5.5), and 8 ml ddH2O) and digested at 37 °C for 35 - 40 min. Then, meristems were washed twice with distilled water and twice with absolute ethanol (5 min each wash). 10 ml of 100% acetic acid was added to each tube, and the root tips were macerated using a homogenizer until a uniform cell suspension was obtained. Microscope slides were placed in a humid chamber, and the cell suspension was dropped onto the slide surface. Slides were incubated in the chamber for 30 min to facilitate chromosome spreading. For FISH, the same procedure was used, and the multiplex oligonucleotide probe ONPM#7 (Chen et al. 2019) (Supplementary Table 1), which contains different 13 oligos that distinguish all wheat chromosomes, was applied.

sgRNA design, cloning and in silico analysis

Following the design guidelines (Schindele et al. 2020), a single sgRNA was designed for each of the following sequences (Supplementary Table 1): the E3900 repeat (GenBank: AF222021.1), the pSc200 repeat (GenBank: Z50039.1), the Bilby repeat (GenBank: AF245032.1), and the single-copy B chromosome-specific gene *DCR400* (Chen et al. 2024). Each sgRNA was subsequently cloned into the generic guide RNA expression vector BSMV-γ-sg, as described (Tamilselvan-Nattar-Amutha et al. 2023). The sgRNAs of pSc200, and Bilby were aligned to the 1RS/1BL reference sequence of the wheat line Zhou8425B (Li et al., 2025) using BLASTn (E-value = 10, maximum target sequences = 10,000) through WheatOmics (Ma et al. 2021) to *in silico* determine their target number. Only alignments with 100% identity over 20 base pairs were considered.

In vitro transcription of the virus and plant inoculation

The *in vitro* transcription of Barley stripe mosaic virus (BSMV) was as described (Wang et al. 2022) with modifications. ~3 - 4 kb DNA fragments of BSMV α, β and γ were amplified using T7 primers (Supplementary Table 1) and ExTaq polymerase (Takara Bio). The PCR products were purified using the Monarch PCR & DNA Cleanup Kit (NEB) and transcribed *in vitro* to α, β and γ virus chains using HiScribe T7 High Yield RNA synthesis kit (New England BioLabs, catalog number E2050S) following the Capped RNA Synthesis protocol provided by the manufacturer. The m7G(5′)ppp(5′)G RNA Cap (New England BioLabs, catalog number S1404L) was used as Cap Analog with 4:1 of Cap Analog: GTP ratio. The quality and concentration of RNA transcripts (usually 2–2.5 µg/µl) were assessed on the agarose gel.

The second leaf of wheat seedlings at the two-leaf stage was inoculated using the mixture of 60 µl phosphate buffer and transcription products of BSMV α, β, and γ chains (2.5 µl each). The phosphate buffer (10 mM) (pH=7.0) contains 0.5% each of celite 545 (Roth) and silicon carbide (400 mesh particle size, Sigma-Aldrich). The inoculation was performed by hand-rubbing the second leaf from the base to the tip while wearing clean nitrile gloves. The procedure was repeated three times for each plant, each time applying

20 µl of the mixture. After inoculation, the plants were kept in the dark overnight. The virus infection symptoms were usually observed 7-10 days after inoculation. The work was performed in a biosafety level 2 (S2) laboratory. The plants used for sequencing and other analyzis were checked by Reverse Transcription-PCR (RT-PCR) using the primer pair gammaF+ gammaR (Supplementary Table 1) to ensure the absence of virus.

Genotyping the chromosomal deletion on the rye B chromosome

After harvesting the seeds from the infected plants, a pair of primers Bnuf2-1 (Supplementary Table 1) targeting the kinetochore nuf2 gene on the B chromosome (Chen et al. 2024) was used to confirm the presence of the rye B chromosome in the progenies. Sc9c130-specific primers were used to confirm the presence of the chromosome end of the rye B chromosome in the progenies (Supplementary Table 1). PCR genotyping was performed using GoTaq DNA Polymerase (Promega) according to the manufacturer’s recommended protocol. PCR products were analyzed on 1% agarose gels. For the single-copy gene Bnuf2-1, PCR amplification was performed with 35 cycles; 25 cycles were used for the high-copy repeat Sc9c130.

Short-read sequencing and mapping

For short-read sequencing, the genomic DNA of plants 1-A10 and 3-D8 carrying a rye B chromosome truncation was sequenced using the DNBSEQ-T7 sequencing platform at BGI Genomics (Hong Kong, China). About 35 Gb paired-end 150 (PE150) data were generated for each sample. Both datasets were aligned to the wheat genome (IWGSC RefSeq v2.1 assembly) plus rye B-pseudomolecule using bowtie2 (v2.5.0, default) (Langmead and Salzberg 2012). The alignments on the B-pseudomolecule and unassigned rye B-like contigs were extracted via samtools (v1.9) (Li et al. 2009) and visualized by pyGenomeTracks (v3.8) (Lopez-Delisle et al. 2021).

Supplementary Table 1

Oligos used in this study

ID	Category	SEQUENCE (5'-3')
Oligo-D1100-1	FISH probe	5′- FITC-ACCGCATCTCCCTCACTCACAATTTCGATTCCTCCTT-3′
Oligo-D1100-2	FISH probe	5′- FITC-GGTCTCGTTTCCCGCCCAAAGTTTCGCCCC-3′
Oligo-D1100-3	FISH probe	5′- FITC-GTATAGCAAAAGAGTTTCCCAAATAGGCGGCACGA-3′
Oligo-D1100-4	FISH probe	5′- FITC-CGGGTATGGGAACGTAGCATGGAGTTTGGTGG-3′
Arabidopsis-type telomere	FISH probe	5′-TAMRA-GGGTTTAGGGTTTAGGGTTT-3′
Oligo-pSc200-6	FISH probe	5'-FAM-CCATACATGGGTGGGGCGCCAAGGACCTGAACACCAAAGTGATATGCCGGCTCATCAAA-3′
Oligo-pSc250	FISH probe	5'-TAMRA-TGTGTTGTTCTTGGACAAAACAATGCATACCATCTCTTCTAC-3'
GAA10	FISH probe/ONPM#7	5′-FAM-GAAGAAGAAGAAGAAGAAGAAGAAGAAGAA-3'
Oligo-pSc119.2-1	FISH probe/ONPM#7	5′-TAMRA (or FAM)-GGCCAGAATCGGCCAAAACTACGAGTGCTGATGACCGACACGTAAACGCACCCCGGGGT-3′
Oligo-pAs1-1	FISH probe/ONPM#7	5′-TAMRA-GGATGCACTTCGTGTACAAAACGGACAATCTCTTTCAAAGTATCAGGATTTCATCC-3′
Oligo-pAs1-3	FISH probe/ONPM#7	5′-TAMRA-TTTTTGTGTGTTCAAAATGCACCATTCAAAGCCACATCATCATTTTTCAATCCTTT-3′
Oligo-pAs1-4	FISH probe/ONPM#7	5′-TAMRA-CTGACTTCATTTGTTATTTTTCATGCATTTACTAATTATTTTTAGCTATAAGACCC-3′
Oligo-pAs1-6	FISH probe/ONPM#7	5′-TAMRA-CATCATTTCATCCACATAGCATGTGCAAGAAAGTTGAGAGGGTTACGGCAAAAACT-3′
Oligo-AFA-3	FISH probe/ONPM#7	5′-TAMRA-AAGTATCAGGGTTTCGGACGGAAACTCATCTATTACAAAGGGATT-3′
Oligo-AFA-4	FISH probe/ONPM#7	5′-TAMRA-CAGTTTTTAAAACATATTTGAACTCCTGACTTTTTGTGTGTT-3′
Oligo-5S-1	FISH probe/ONPM#7	5′-TAMRA-TCATACCAGCACTAAAGCACCGGATCCCATCAGAAC-3′
Oligo-5S-2	FISH probe/ONPM#7	5′-TAMRA-GCGTGCTTGGGCGAGAGTAGTACTAGGATGGGTGAC-3′
Oligo-BSCL135-1	FISH probe/ONPM#7	5′-FAM-GCCTCGACTCGCGTTACCCTAAGATAGACAG-3′
Oligo-BSCL135-2	FISH probe/ONPM#7	5′-FAM-GTGGTGTCACGTGCTCGTCTCGGCGTCCGT-3′
Oligo-BSCL242-1	FISH probe/ONPM#7	5′-FAM-GTTGCTCAAACTAGAAAATGGACAGATATGGT-3′

Continue: Supplementary Table 1 Oligos used in this study

ID	Category	SEQUENCE (5'-3')
NCR400_g1F	sgRNA cloning	GTAAGAAGGAAACCAGGAAGAAGTGTTT
NCR400_g1R	sgRNA cloning	TCTAAAACACTTCTTCCTGGTTTCCTTC
gBilby-2F	sgRNA cloning	GTAAGGGGTGAAGGAGGGTCCATGGTTT
gBilby-2R	sgRNA cloning	TCTAAAACCATGGACCCTCCTTCACCCC
gE3900-6F	sgRNA cloning	GTAACAGGTGATGGATCGAGACAGGTTT
gE3900-6R	sgRNA cloning	TCTAAAACCTGTCTCGATCCATCACCTG
gpSc200-1F	sgRNA cloning	GTAATAGGCGTTGGAAAGCTATTGGTTT
gpSc200-1R	sgRNA cloning	TCTAAAACCAATAGCTTTCCAACGCCTA
gCenTt566-7F	sgRNA cloning	GTAATACTCCCCGCTTGCGCCAGGGTTT
gCenTt566-7R	sgRNA cloning	TCTAAAACCCTGGCGCAAGCGGGGAGTA
zCAS9-F	PCR primer	CGGACTAGTATGGATTACAAGGACCACGACG
zCAS9seq1	PCR primer	ACCTTGTACTCGTCGGTGATCAC
Sc9c130-fwd	PCR primer	GCATGTCATCGGTAGGATAGG
Sc9c130-rev	PCR primer	ACCCCTTCCCTTTCGATCTAC
Bnuf2-1F	PCR primer	TGGTTCCTCTCGCTTGTTCC
Bnuf2-1R	PCR primer	GGCGAGTGTGAAGTCCTTGA
[1]BSMValpha_T7_F	T7 primer	TAATACGACTCACTATAGTATGTAAGTTGCCTTTGGG
[1]BSMVbeta_T7_F	T7 primer	TAATACGACTCACTATAGTAAAAGAAAAGGAACAACC
[1]BSMVgamma_T7_F	T7 primer	TAATACGACTCACTATAGTATAGCTTGAGCATTACCG
[1]BSMVcommon_R	T7 primer	TGGTCTTCCCTTGGGGGACC
gammaF	Virus-detection	TGGCTAAGCTTGAAAGTGAGG
gammaR	Virus-detection	TAAAGTGTGACGCAGCTACC

1: amplification of α, β, and γ are using their corresponding left primers and the common right primer BSMVcommon_R.

Supplementary Table 2

B chromosome truncations detected by PCR

sgRNA target	Screened plants	B-positive plants [a]	Plants with B truncation	Frequency of B truncations [b]
E3900	179	82	8	9.8%
DCR400	182	86	0	0%

a Only a subset of the plants carried the B chromosome, and those selected for VIGE contained only a single rye B chromosome. To identify the presence of the rye B chromosome, we used primers (Bnuf2) targeting the rye B-specific variant of the kinetochore protein nuf2 (321,949,858-321,954,295 bp on the assembled B sequence).

b The frequency of induced truncations = (Plants with B-deletion / B-positive plants)

Supplementary Table 3

Statistics of CRISPR/Cas9-mediated ‘cutting’ of pSc200 and Bilby repeats on 1RS

Number of screened plants for cutting pSc200	Plants with reduced pSc200 signals	Plants with complete loss of the pSc200 FISH signal
18	17 (94.4%)	3 (16.7%)

Number of screened plants for cutting Bilby	Plants with monosomic 1RS/1BL	Plants with monosomic 1RS/1RS
16	4 (25%)	1 (6.3%)

Supplementary Table 4

FISH result of the M_1 plants from CRISPR-based 'cutting of pSc200

Plant name	pSc200 homolog chromosome I	pSc200 homolog chromosome II	Note
Parent	+	+	normal
V1-1	+	-	reduction
V1-2	+	-	reduction
V1-3	+	lost	heterozygous deletion
V1-4	+	-	reduction
V2-1	+	-	reduction
V2-2	+	+	normal
V2-3	+	-	reduction
V2-4	+	-	reduction
V2-5	-	lost	heterozygous deletion
V2-6	lost	lost	homozygous deletion
V2-7	-	lost	heterozygous deletion
V2-8	-	lost	heterozygous deletion
V2-9	+	-	reduction
V3-1	lost	lost	homozygous deletion
V3-2	-	-	reduction
V3-3	lost	lost	homozygous deletion
V3-4	-	lost	heterozygous deletion
V3-5	+	lost	heterozygous deletion

Note: '+' indicates FISH signals similar to the wild-type parent. '-' indicates signals substantially reduced compared to the wild-type parent. 'lost' indicates no pSc200-signal.

Supplementary Table 5

Analysis of the M_1 plants from CRISPR/Cas9-based ‘cutting of the Bilby repeat. Bilby and pSc200s-specific probes were used for FISH.

Plant name	FISH result
Parent	Disomic 1RS/1BL
035	Monosomic 1RS/1BL
032	Monosomic 1RS/1BL
034	Monosomic 1RS/1BL
0314	Monosomic 1RS/1BL
0311	Monosomic 1RS/1BL, monosomic 1RS/1RS, and monosomic 1BL/1BL
143	Disomic 1RS/1BL
031	Disomic 1RS/1BL
033	Disomic 1RS/1BL
141	Disomic 1RS/1BL
142	Disomic 1RS/1BL
041	Disomic 1RS/1BL
042	Disomic 1RS/1BL
044	Disomic 1RS/1BL
045	Disomic 1RS/1BL
0312	Disomic 1RS/1BL
0313	Disomic 1RS/1BL

Supplementary Table 6

Genome-wide prediction of target sites for sgRNAs used in this study

Reference

Adikusuma F, Williams N, Grutzner F, Hughes J, Thomas P (2017) Targeted Deletion of an Entire Chromosome Using CRISPR/Cas9. Molecular therapy : the journal of the American Society of Gene Therapy. doi:10.1016/j.ymthe.2017.05.021

Aliyeva-Schnorr L, Beier S, Karafiátová M, Schmutzer T, Scholz U, Doležel J, Stein N, Houben A (2015) Cytogenetic mapping with centromeric bacterial artificial chromosomes contigs shows that this recombination-poor region comprises more than half of barley chromosome 3 H. The Plant Journal 84 (2):385-394

Anton T, Bultmann S, Leonhardt H, Markaki Y (2014) Visualization of specific DNA sequences in living mouse embryonic stem cells with a programmable fluorescent CRISPR/Cas system. Nucleus 5 (2):163-172. doi:10.4161/nucl.28488

Birchler JA, Kelly J, Singh J, Liu H, Zhang Z, Char SN, Sharma M, Yang H, Albert PS, Yang B (2024) Synthetic minichromosomes in plants: past, present, and promise. The Plant Journal 120 (6):2356-2366

Blunden R, Wilkes TJ, Forster JW, Jimenez MM, Sandery MJ, Karp A, Jones RN (1993) Identification of the E3900 family, a 2nd family of rye chromosome-B specific repeated sequences. Genome / National Research Council Canada = Genome / Conseil national de recherches Canada 36 (4):706-711

Chen J, Bartoš J, Boudichevskaia A, Voigt A, Rabanus-Wallace MT, Dreissig S, Tulpová Z, Šimková H, Macas J, Kim G, ... & Houben, A. (2024) The genetic mechanism of B chromosome drive in rye illuminated by chromosome-scale assembly. Nature Communications 15 (1):9686

Chen J, Tang Y, Yao L, Wu H, Tu X, Zhuang L, Qi Z (2019) Cytological and molecular characterization of Thinopyrum bessarabicum chromosomes and structural rearrangements introgressed in wheat. Molecular Breeding 39 (10):1-14

Daer RM, Cutts JP, Brafman DA, Haynes KA (2017) The Impact of Chromatin Dynamics on Cas9-Mediated Genome Editing in Human Cells. Acs Synth Biol 6 (3):428-438. doi:10.1021/acssynbio.5b00299

Deng WL, Shi XH, Tjian R, Lionnet T, Singer RH (2015) CASFISH: CRISPR/Cas9-mediated in situ labeling of genomic loci in fixed cells. P Natl Acad Sci USA 112 (38):11870-11875. doi:10.1073/pnas.1515692112

Dreissig S, Schiml S, Schindele P, Weiss O, Rutten T, Schubert V, Gladilin E, Mette MF, Puchta H, Houben A (2017) Live-cell CRISPR imaging in plants reveals dynamic telomere movements. Plant J 91 (4):565-573. doi:10.1111/tpj.13601

Endo T, Gill B (1996) The deletion stocks of common wheat. Journal of Heredity 87 (4):295-307

Endo TR, Nasuda S, Jones N, Dou Q, Akahori A, Wakimoto M, Tanaka H, Niwa K, Tsujimoto H (2008) Dissection of rye B chromosomes, and nondisjunction properties of the dissected segments in a common wheat background. Genes & genetic systems 83 (1):23-30

Francki MG (2001) Identification of Bilby, a diverged centromeric Ty1-copia retrotransposon family from cereal rye (Secale cereale L.). Genome / National Research Council Canada = Genome / Conseil national de recherches Canada 44 (2):266-274

Fu S, Chen L, Wang Y, Li M, Yang Z, Qiu L, Yan B, Ren Z, Tang Z (2015) Oligonucleotide probes for ND-FISH analysis to identify rye and wheat chromosomes. Scientific Reports 5 (1):10552

Gehrke F, Ruiz-Duarte P, Schindele A, Wolf S, Puchta H (2023) An inducible CRISPR-Kill system for temporally controlled cell type-specific cell ablation in Arabidopsis thaliana. New Phytol 239 (5):2041-2052. doi:10.1111/nph.19102

Guo J, Lei Y, Zhang H, Song D, Liu X, Cao Z, Chu C, Zhuang L, Qi Z (2019) Frequent variations in tandem repeats pSc200 and pSc119. 2 cause rapid chromosome evolution of open-pollinated rye. Molecular Breeding 39:1-13

Hashizume R, Wakita S, Sawada H, Takebayashi SI, Kitabatake Y, Miyagawa Y, Hirokawa YS, Imai H, Kurahashi H (2025) Trisomic rescue via allele-specific multiple chromosome cleavage using CRISPR-Cas9 in trisomy 21 cells. PNAS Nexus 4 (2):pgaf022. doi:10.1093/pnasnexus/pgaf022

Huang X, Zhu M, Zhuang L, Zhang S, Wang J, Chen X, Wang D, Chen J, Bao Y, Guo J (2018) Structural chromosome rearrangements and polymorphisms identified in Chinese wheat cultivars by high-resolution multiplex oligonucleotide FISH. Theoretical and Applied Genetics 131:1967-1986

Ishii T, Schubert V, Khosravi S, Dreissig S, Metje-Sprink J, Sprink T, Fuchs J, Meister A, Houben A (2019) RNA-guided endonuclease - in situ labelling (RGEN-ISL): a fast CRISPR/Cas9-based method to label genomic sequences in various species. New Phytol 222 (3):1652-1661. doi:10.1111/nph.15720

Kapusi E, Ma L, Teo CH, Hensel G, Himmelbach A, Schubert I, Mette MF, Kumlehn J, Houben A (2012) Telomere-mediated truncation of barley chromosomes. Chromosoma 121:181-190

Langmead B, Salzberg SL (2012) Fast gapped-read alignment with Bowtie 2. Nature methods 9 (4):357-359

Li H, Handsaker B, Wysoker A, Fennell T, Ruan J, Homer N, Marth G, Abecasis G, Durbin R, Subgroup GPDP (2009) The sequence alignment/map format and SAMtools. bioinformatics 25 (16):2078-2079

Lopez-Delisle L, Rabbani L, Wolff J, Bhardwaj V, Backofen R, Grüning B, Ramírez F, Manke T (2021) pyGenomeTracks: reproducible plots for multivariate genomic datasets. Bioinformatics 37 (3):422-423

Lukaszewski AJ (1997) Construction of midget chromosomes in wheat. Genome 40 (4):566-569

Ma S, Wang M, Wu J, Guo W, Chen Y, Li G, Wang Y, Shi W, Xia G, Fu D (2021) WheatOmics: A platform combining multiple omics data to accelerate functional genomics studies in wheat. Molecular Plant 14 (12):1965-1968

Nelson AD, Lamb JC, Kobrossly PS, Shippen DE (2011) Parameters affecting telomere-mediated chromosomal truncation in Arabidopsis. The Plant Cell 23 (6):2263-2272

Potlapalli BP, Dassau F, Fuchs J, Sushmoy DR, Houben A (2025) CRISPR-CISH: an in situ chromogenic DNA repeat detection system for research and life science education. Chromosome Res 33 (1):7. doi:10.1007/s10577-025-09767-1

Potlapalli BP, Fuchs J, Rutten T, Meister A, Houben A (2024) The potential of ALFA-tag and tyramide-based fluorescence signal amplification to expand the CRISPR-based DNA imaging toolkit. J Exp Bot 75 (20):6244-6257. doi:10.1093/jxb/erae341

Potlapalli BP, Schubert V, Metje-Sprink J, Liehr T, Houben A (2020) Application of Tris-HCl allows the specific labeling of regularly prepared chromosomes by CRISPR-FISH. Cytogenet Genome Res 160 (3):156-165. doi:10.1159/000506720

Puchta H, Houben A (2024) Plant chromosome engineering - past, present and future. New Phytol 241 (2):541-552. doi:10.1111/nph.19414

Riley R, Chapman V (1958) Genetic control of the cytologically diploid behaviour of hexaploid wheat.

Samach A, Mafessoni F, Gross O, Melamed-Bessudo C, Filler-Hayut S, Dahan-Meir T, Amsellem Z, Pawlowski WP, Levy AA (2023) CRISPR/Cas9-induced DNA breaks trigger crossover, chromosomal loss, and chromothripsis-like rearrangements. Plant Cell. doi:10.1093/plcell/koad209

Sandery MJ, Forster JW, Blunden R, Jones RN (1990a) Identification of a family of repeated sequences on the rye B-chromosome. Genome / National Research Council Canada = Genome / Conseil national de recherches Canada 33 (6):908-913

Sandery MJ, Forster JW, Blunden R, Jones RN (1990b) Identification of a family of repeated sequences on the rye B chromosome. Genome / National Research Council Canada = Genome / Conseil national de recherches Canada 33 (6):908-913

Schindele A, Gehrke F, Schmidt C, Rohrig S, Dorn A, Puchta H (2022) Using CRISPR-Kill for organ specific cell elimination by cleavage of tandem repeats. Nat Commun 13 (1):1502. doi:10.1038/s41467-022-29130-w

Schindele P, Wolter F, Puchta H (2020) CRISPR guide RNA design guidelines for efficient genome editing. RNA Tagging: methods and protocols:331-342

Schubert I, Oud JL (1997) There is an upper limit of chromosome size for normal development of an organism. Cell 88 (4):515-520

Schubert I, Rieger R (1985) A new mechanism for altering chromosome number during karyotype evolution. Theor Appl Genet 70 (2):213-221. doi:10.1007/BF00275324

Sears E (1956) The transfer of leaf-rust resistance from Aegilops umbellulata to wheat.

Sears E, Okamoto M (1958) Intergenomic chromosome relationships in hexaploid wheat.

Sears ER (1954) The aneuploids of common wheat. University of Missouri, College of Agriculture, Agricultural Experiment Station,

Shi F, Endo TR (1997) Production of wheat-barley disomic addition lines possessing an Aegilops cylindrica gametocidal chromosome. Genes & Genetic Systems 72 (4):243-248

Tamilselvan-Nattar-Amutha S, Dreissig S, Kumlehn J, Heckmann S (2023) Barley stripe mosaic virus-mediated somatic and heritable gene editing in barley (Hordeum vulgare L.). Frontiers in Plant Science 14:1201446

Teo CH, Ma L, Kapusi E, Hensel G, Kumlehn J, Schubert I, Houben A, Mette MF (2011) Induction of telomere-mediated chromosomal truncation and stability of truncated chromosomes in Arabidopsis thaliana. The Plant Journal 68 (1):28-39

Tomita M, Shinohara K, Morimoto M (2008) Revolver is a new class of transposon-like gene composing the Triticeae genome. DNA research 15 (1):49-62

Vershinin AV, Schwarzacher T, Heslop-Harrison JS (1995) The large-scale genomic organization of repetitive DNA families at the telomeres of rye chromosomes. The Plant Cell 7 (11):1823-1833

Wang W, Yu Z, He F, Bai G, Trick HN, Akhunova A, Akhunov E (2022) Multiplexed promoter and gene editing in wheat using a virus-based guide RNA delivery system. Plant Biotechnology Journal 20 (12):2332-2341

Weiss T, Crisp PA, Rai KM, Song M, Springer NM, Zhang F (2022) Epigenetic features drastically impact CRISPR-Cas9 efficacy in plants. Plant Physiol 190 (2):1153-1164. doi:10.1093/plphys/kiac285

Xu C, Cheng Z, Yu W (2012) Construction of rice mini-chromosomes by telomere-mediated chromosomal truncation. The Plant Journal 70 (6):1070-1079

Yan X, Li C, Yang J, Wang L, Jiang C, Wei W (2017) Induction of telomere-mediated chromosomal truncation and behavior of truncated chromosomes in Brassica napus. The Plant Journal 91 (4):700-713

Yu W, Han F, Gao Z, Vega JM, Birchler JA (2007) Construction and behavior of engineered minichromosomes in maize. Proceedings of the National Academy of Sciences 104 (21):8924-8929

Yu W, Lamb JC, Han F, Birchler JA (2006) Telomere-mediated chromosomal truncation in maize. Proceedings of the National Academy of Sciences 103 (46):17331-17336

Yuan J, Shi Q, Guo X, Liu Y, Su H, Guo X, Lv Z, Han F (2017) Site-specific transfer of chromosomal segments and genes in wheat engineered chromosomes. Journal of Genetics and Genomics 44 (11):531-539

Zhao Y, Huang Z, Zhou X, Teng W, Liu Z, Wang W, Tang S, Liu Y, Liu J, Wang W (2025) Precise deletion, replacement and inversion of large DNA fragments in plants using dual prime editing. Nature Plants:1-15
Zuo E, Huo X, Yao X, Hu X, Sun Y, Yin J, He B, Wang X, Shi L, Ping J, Wei Y, Ying W, Wei W, Liu W, Tang C, Li Y, Hu J, Yang H (2017) CRISPR/Cas9-mediated targeted chromosome elimination. Genome Biol 18 (1):224. doi:10.1186/s13059-017-1354-4
