# Supplementary material for: Satellite DNA-targeted CRISPR–Cas9-mediated editing enables chromosome truncation and elimination in wheat
Source: Plant Commun. 2026 Mar 23;7(7):101833. doi: 10.1016/j.xplc.2026.101833 (PMC13370215; doi:10.1016/j.xplc.2026.101833)
Supplement: Document S2. Supplemental Figures S1–S8 [file mmc2.pptx]

## Slide 1
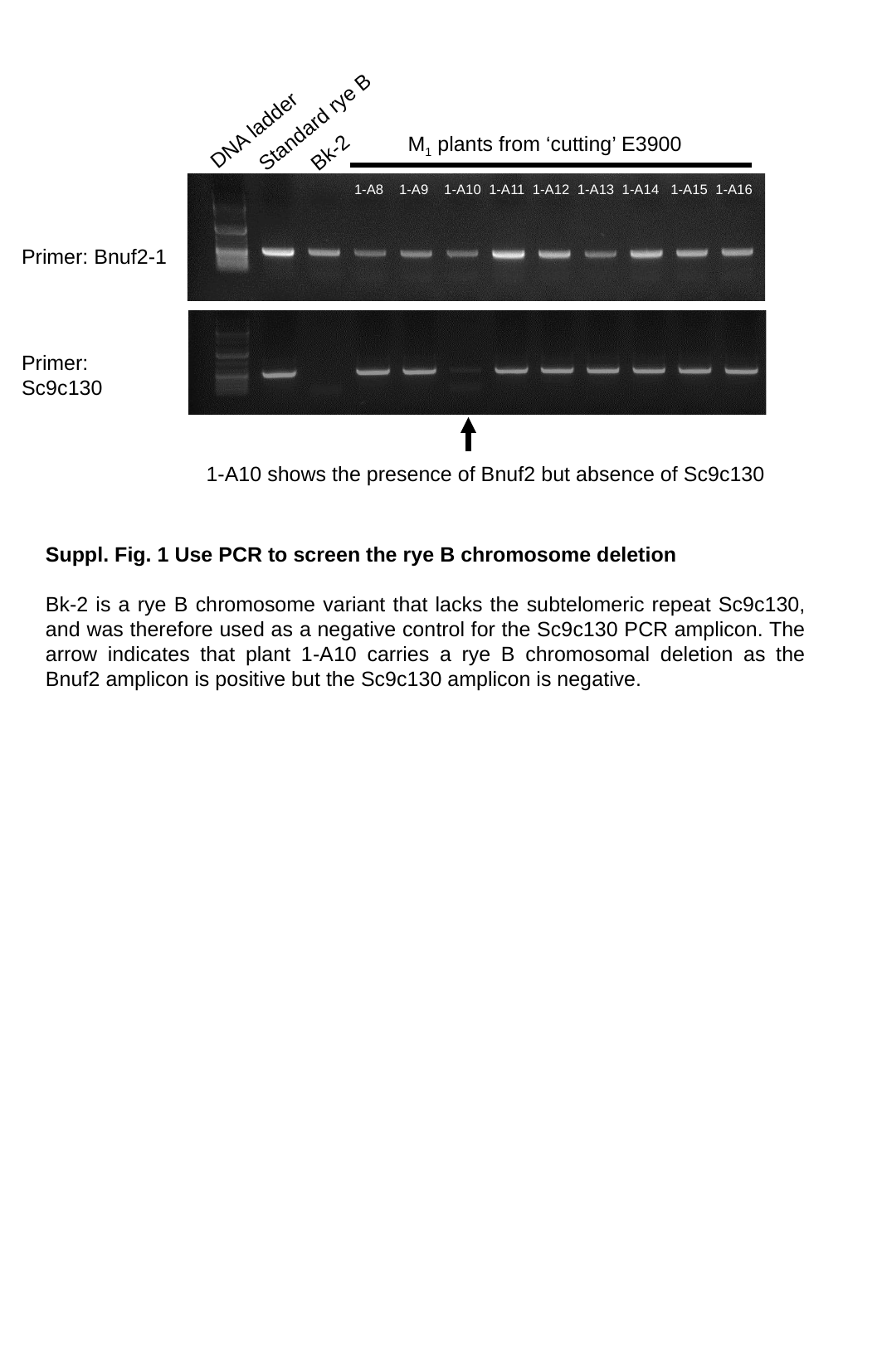

DNA ladder
Standard rye B
Bk-2
M1 plants from ‘cutting’ E3900
1-A8 1-A9 1-A10 1-A11 1-A12 1-A13 1-A14 1-A15 1-A16
Primer: Bnuf2-1
Primer: Sc9c130
1-A10 shows the presence of Bnuf2 but absence of Sc9c130
Suppl. Fig. 1 Use PCR to screen the rye B chromosome deletion
Bk-2 is a rye B chromosome variant that lacks the subtelomeric repeat Sc9c130, and was therefore used as a negative control for the Sc9c130 PCR amplicon. The arrow indicates that plant 1-A10 carries a rye B chromosomal deletion as the Bnuf2 amplicon is positive but the Sc9c130 amplicon is negative.

## Slide 2
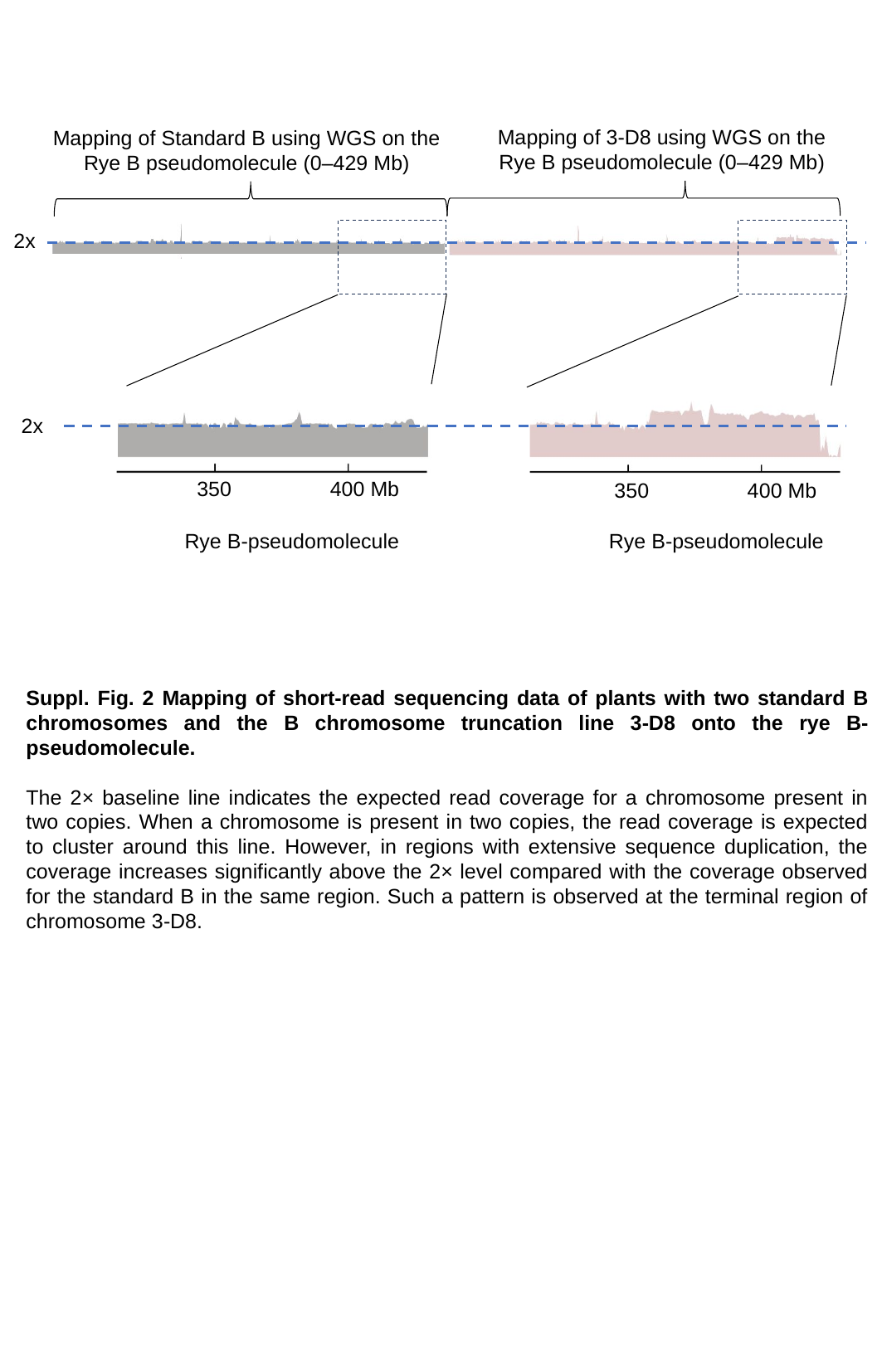

Mapping of 3-D8 using WGS on the Rye B pseudomolecule (0–429 Mb)
Mapping of Standard B using WGS on the Rye B pseudomolecule (0–429 Mb)
2x
2x
350 400 Mb
350 400 Mb
Rye B-pseudomolecule
Rye B-pseudomolecule
Suppl. Fig. 2 Mapping of short-read sequencing data of plants with two standard B chromosomes and the B chromosome truncation line 3-D8 onto the rye B-pseudomolecule.
The 2× baseline line indicates the expected read coverage for a chromosome present in two copies. When a chromosome is present in two copies, the read coverage is expected to cluster around this line. However, in regions with extensive sequence duplication, the coverage increases significantly above the 2× level compared with the coverage observed for the standard B in the same region. Such a pattern is observed at the terminal region of chromosome 3-D8.

## Slide 3
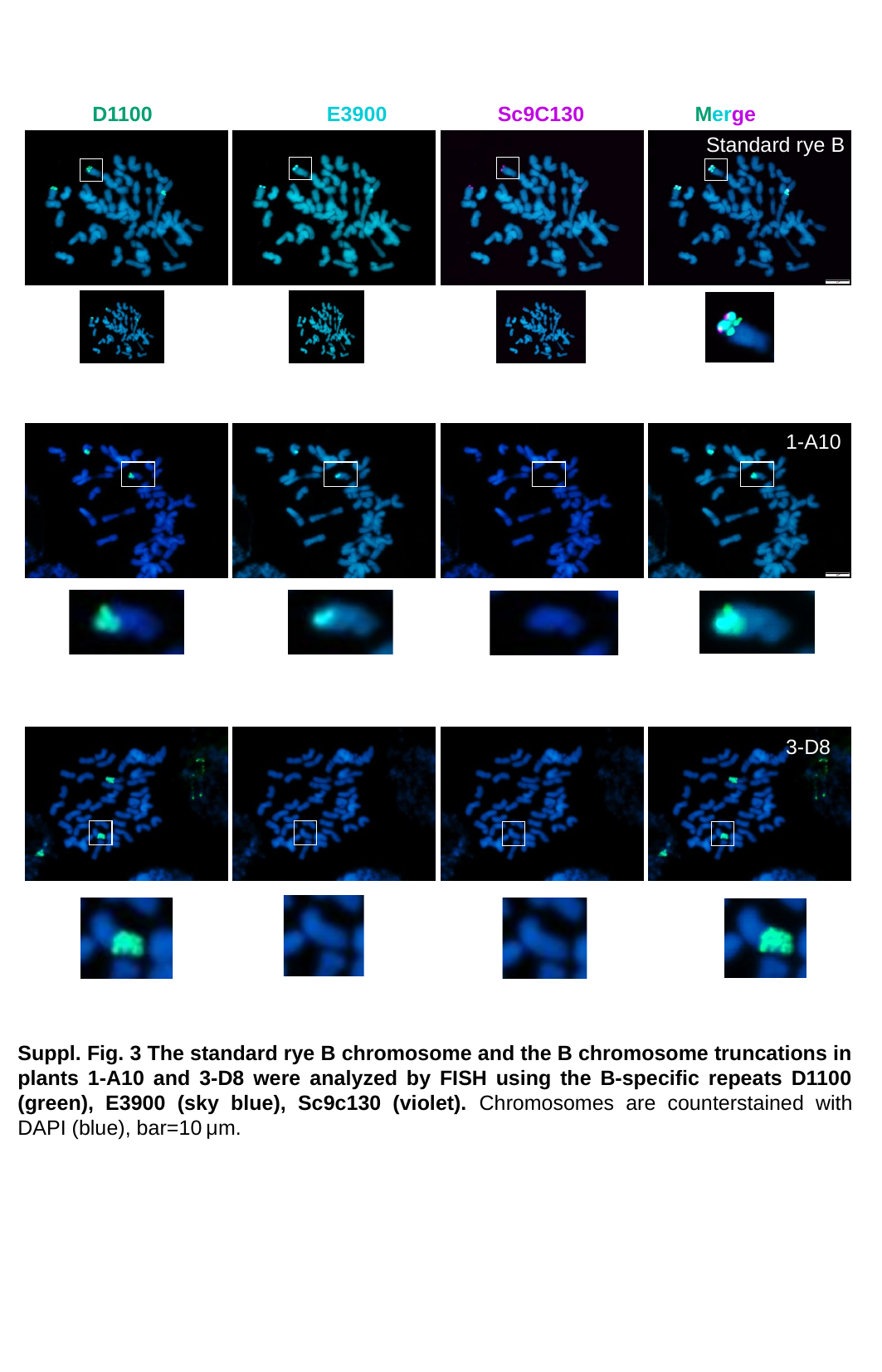

D1100 E3900 Sc9C130 Merge
Standard rye B
1-A10
3-D8
Suppl. Fig. 3 The standard rye B chromosome and the B chromosome truncations in plants 1-A10 and 3-D8 were analyzed by FISH using the B-specific repeats D1100 (green), E3900 (sky blue), Sc9c130 (violet). Chromosomes are counterstained with DAPI (blue), bar=10 μm.

## Slide 4
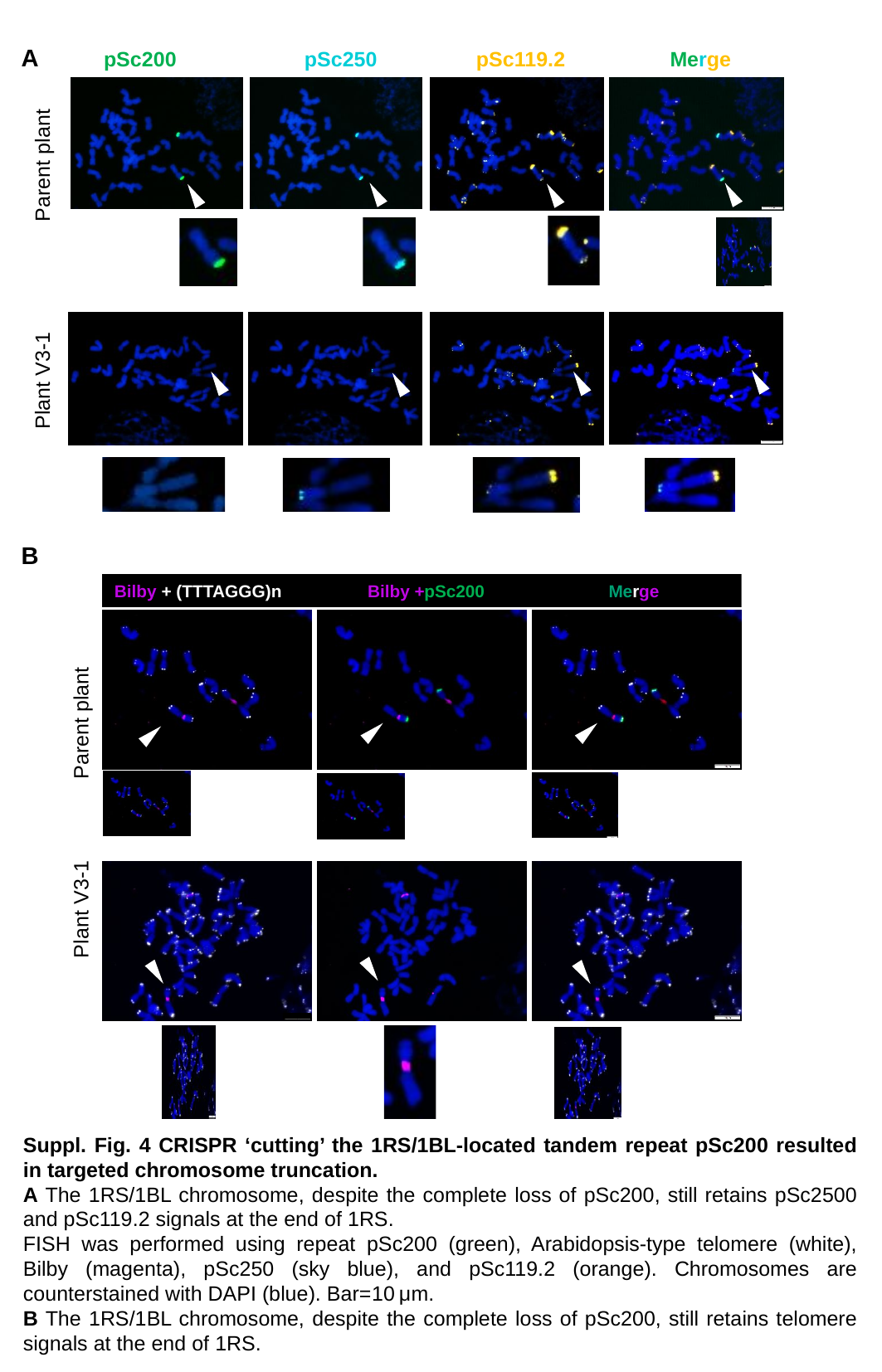

A
pSc200 pSc250 pSc119.2 Merge
Plant V3-1 Parent plant
B
Bilby + (TTTAGGG)n Bilby +pSc200 Merge
Plant V3-1 Parent plant
Suppl. Fig. 4 CRISPR ‘cutting’ the 1RS/1BL-located tandem repeat pSc200 resulted in targeted chromosome truncation.
A The 1RS/1BL chromosome, despite the complete loss of pSc200, still retains pSc2500 and pSc119.2 signals at the end of 1RS.
FISH was performed using repeat pSc200 (green), Arabidopsis-type telomere (white), Bilby (magenta), pSc250 (sky blue), and pSc119.2 (orange). Chromosomes are counterstained with DAPI (blue). Bar=10 μm.
B The 1RS/1BL chromosome, despite the complete loss of pSc200, still retains telomere signals at the end of 1RS.

## Slide 5
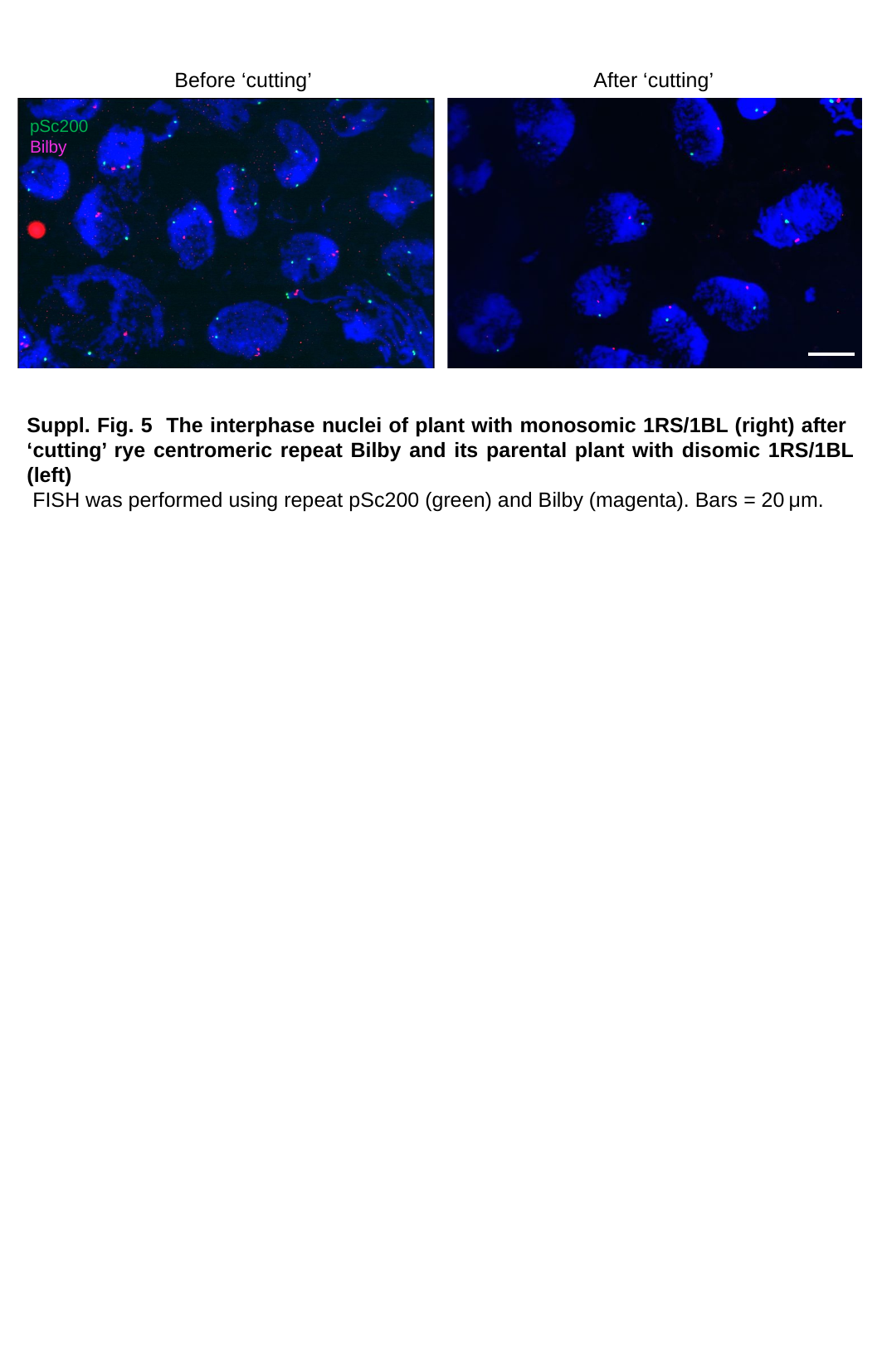

Before ‘cutting’ After ‘cutting’
pSc200
Bilby
Suppl. Fig. 5 The interphase nuclei of plant with monosomic 1RS/1BL (right) after ‘cutting’ rye centromeric repeat Bilby and its parental plant with disomic 1RS/1BL (left)
 FISH was performed using repeat pSc200 (green) and Bilby (magenta). Bars = 20 μm.

## Slide 6
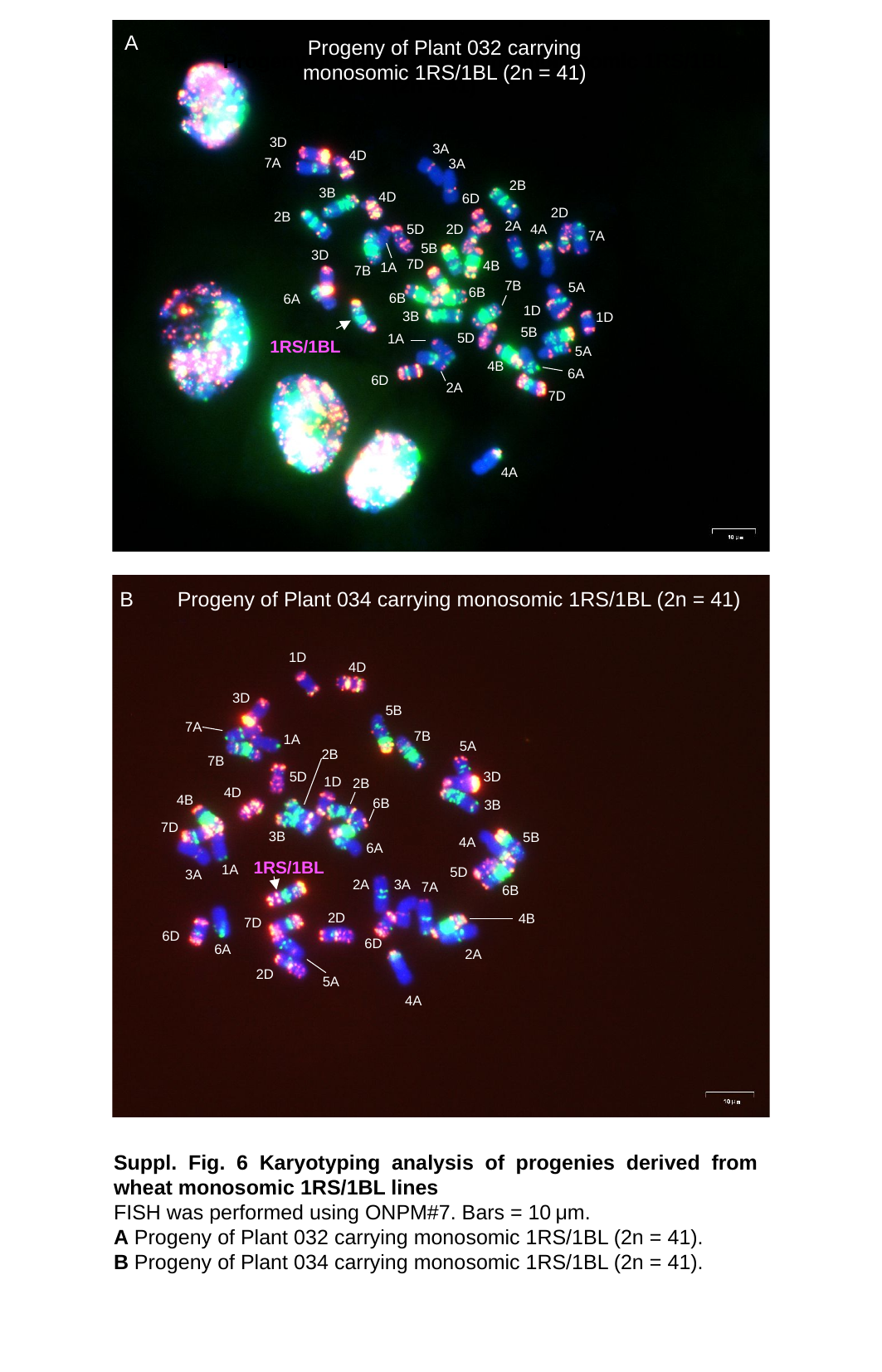

A
Progeny of Plant 032 carrying monosomic 1RS/1BL (2n = 41)
 Progeny of Plant 032 carrying monosomic 1RS/1BL
(2n = 41)
3D
3A
4D
7A
3A
2B
3B
4D
6D
2D
2B
2A
5D
4A
2D
7A
5B
3D
7D
4B
1A
7B
7B
5A
6B
6B
6A
1D
3B
1D
5B
5D
1A
1RS/1BL
5A
4B
6A
6D
2A
7D
4A
Progeny of Plant 034 carrying monosomic 1RS/1BL (2n = 41)
B
1D
4D
3D
5B
7A
7B
1A
5A
2B
7B
5D
3D
1D
2B
4D
4B
6B
3B
7D
3B
5B
4A
6A
1RS/1BL
1A
5D
3A
2A
3A
7A
6B
2D
4B
7D
6D
6D
6A
2A
2D
5A
4A
Suppl. Fig. 6 Karyotyping analysis of progenies derived from wheat monosomic 1RS/1BL lines
FISH was performed using ONPM#7. Bars = 10 μm.
A Progeny of Plant 032 carrying monosomic 1RS/1BL (2n = 41).
B Progeny of Plant 034 carrying monosomic 1RS/1BL (2n = 41).

## Slide 7
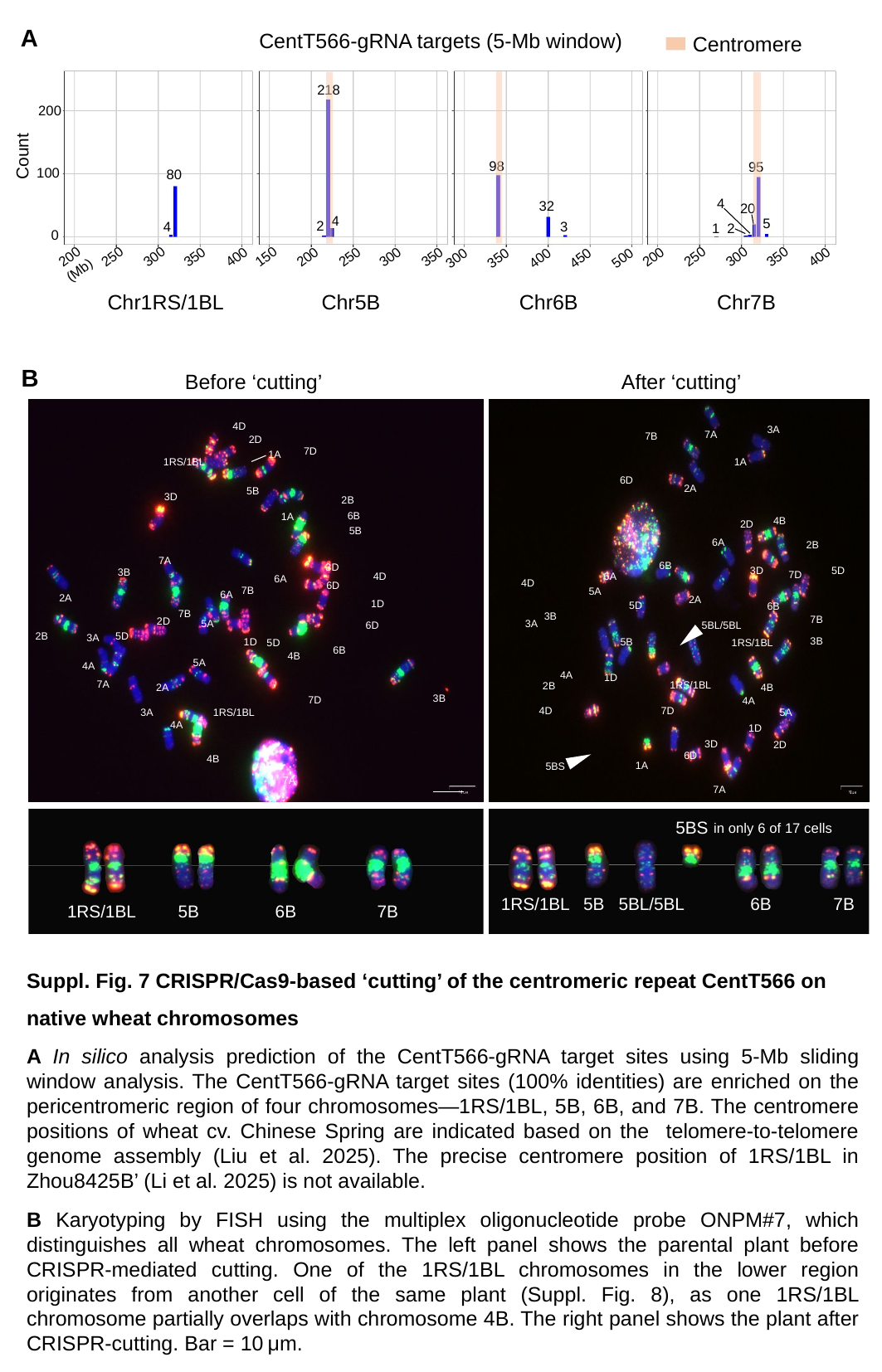

A
CentT566-gRNA targets (5-Mb window)
Centromere
218
200
Count
98
95
100
80
4
32
 20
4
5
2
3
4
1
 2
0
200
(Mb)
200
300
150
250
250
350
400
200
300
350
300
250
350
400
300
400
350
450
500
Chr1RS/1BL Chr5B Chr6B Chr7B
B
Before ‘cutting’ After ‘cutting’
4D
3A
7A
7B
2D
7D
1A
1RS/1BL
1A
6D
2A
5B
3D
2B
6B
1A
4B
2D
5B
6A
2B
7A
6B
3D
3D
5D
3B
7D
6A
4D
6A
4D
6D
7B
5A
6A
2A
2A
1D
5D
6B
7B
3B
7B
2D
5A
3A
6D
5BL/5BL
2B
5D
3A
3B
5B
1D
1RS/1BL
5D
6B
4B
5A
4A
4A
1D
7A
1RS/1BL
2B
4B
2A
3B
7D
4A
7D
4D
1RS/1BL
5A
3A
4A
1D
3D
2D
6D
4B
1A
5BS
7A
7A
5BS
in only 6 of 17 cells
1RS/1BL 5B 5BL/5BL 6B 7B
1RS/1BL 5B 6B 7B
Suppl. Fig. 7 CRISPR/Cas9-based ‘cutting’ of the centromeric repeat CentT566 on native wheat chromosomes
A In silico analysis prediction of the CentT566-gRNA target sites using 5-Mb sliding window analysis. The CentT566-gRNA target sites (100% identities) are enriched on the pericentromeric region of four chromosomes—1RS/1BL, 5B, 6B, and 7B. The centromere positions of wheat cv. Chinese Spring are indicated based on the telomere-to-telomere genome assembly (Liu et al. 2025). The precise centromere position of 1RS/1BL in Zhou8425B’ (Li et al. 2025) is not available.
B Karyotyping by FISH using the multiplex oligonucleotide probe ONPM#7, which distinguishes all wheat chromosomes. The left panel shows the parental plant before CRISPR-mediated cutting. One of the 1RS/1BL chromosomes in the lower region originates from another cell of the same plant (Suppl. Fig. 8), as one 1RS/1BL chromosome partially overlaps with chromosome 4B. The right panel shows the plant after CRISPR-cutting. Bar = 10 μm.

## Slide 8
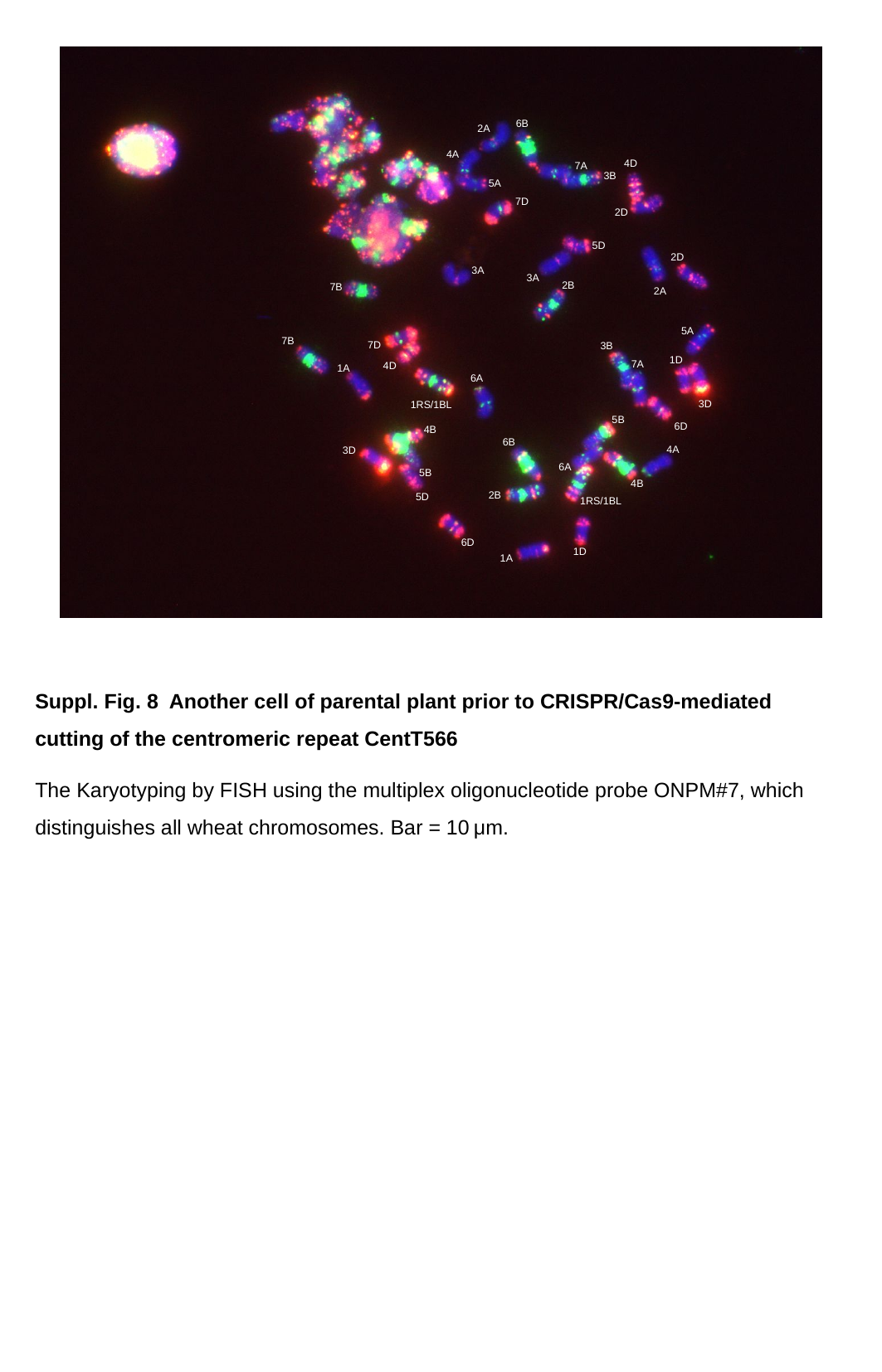

6B
2A
4A
4D
7A
3B
5A
7D
2D
5D
2D
3A
3A
2B
7B
2A
5A
7B
7D
3B
1D
7A
4D
1A
6A
3D
1RS/1BL
5B
6D
4B
6B
4A
3D
6A
5B
4B
2B
5D
6D
1D
1A
Suppl. Fig. 8 Another cell of parental plant prior to CRISPR/Cas9-mediated cutting of the centromeric repeat CentT566
The Karyotyping by FISH using the multiplex oligonucleotide probe ONPM#7, which distinguishes all wheat chromosomes. Bar = 10 μm.
1RS/1BL
