# Supplementary material for: Satellite DNA-targeted CRISPR–Cas9-mediated editing enables chromosome truncation and elimination in wheat
Source: Plant Commun. 2026 Mar 23;7(7):101833. doi: 10.1016/j.xplc.2026.101833 (PMC13370215; doi:10.1016/j.xplc.2026.101833)
Supplement: Document S3. Article plus supplemental information [file mmc4.pdf]

Plant Communications
Correspondence

CellPress
Partner Journal

Satellite DNA-targeted CRISPR–Cas9-mediated editing enables chromosome truncation and elimination in wheat

Dear Editor,

Although CRISPR–Cas has advanced chromosome engineering in model systems such as *Arabidopsis* (Yasar et al., 2026), manipulation of large-genome crops remains challenging (Supplemental Note 1). Here, we present virus-induced genome editing (VIGE) using satellite repeat-specific single guide RNAs (sgRNAs) as a tool for chromosomal alterations. To minimize lethality caused by genetic loss, we first performed *in planta* chromosome truncation in a wheat–rye B chromosome addition line. The Cas9-expressing line 707 of wheat cv. "Bobwhite" (Wang et al., 2022) was used as a pollinator in a cross with a wheat "Chinese Spring" line carrying the dispensable rye B chromosome (Endo et al., 2008; Chen et al., 2024). B chromosome-positive (B) and Cas9-positive plants were identified using the B-specific satellite D1100 as a fluorescence *in situ* hybridization (FISH) probe and a Cas9-specific primer pair (Supplemental Table 1). Cas9-positive plants carrying a single B were selected to avoid post-editing heterozygosity of the B chromosome. Two sgRNAs were designed (Supplemental Table 1): one targeting the B-specific satellite E3900 and the other targeting the B-specific single-copy gene *DCR400* (Chen et al., 2024) as a control (Figure 1A).

Both gRNAs were delivered via Barley stripe mosaic virus (BSMV) (Tamilselvan-Nattar-Amutha et al., 2023) to five Cas9- and B-positive F_1 plants each. This generation was designated M_0, as genome editing is initiated upon the combination of sgRNA and Cas9. Only the resulting virus-free M_1 plants were subsequently analyzed, as the absence of the virus prevents further editing activity. The M_1 plants were genotyped using a primer pair specific for the B-specific gene *Bnuf2* (Figure 1A and Supplemental Table 1). A primer pair specific for the subtelomeric B-specific repeat Sc9c130 (Supplemental Table 1) was used to identify truncations of the long arm of the B chromosome (Figure 1A), as CRISPR-induced breaks within the E3900 array may delete the region distal to the break. In the experiment targeting repeat Sc9c130, 82 of 179 PCR-genotyped plants contained a B chromosome, of which eight (9.8%) were negative for Sc9c130 (Supplemental Figure 1). In contrast, in the experiment targeting the single-copy gene *DCR400,* none of the 86 B-containing plants showed a terminal deletion (Supplemental Table 2).

To validate the loss of the Sc9c130 repeat, we randomly selected two RT–PCR-confirmed, virus-free plants, 1-A10 and 3-D8, carrying a Sc9c130-free B chromosome for whole-genome short-read sequencing (WGS) at approximately 2-fold genome coverage. The reads were aligned to the rye B reference sequence, together with WGS data from wheat plants carrying two standard B chromosomes as a control (Figure 1B). Significantly reduced read mapping was observed in 1-A10 and 3-D8 at the distal 424–429 Mb region, corresponding to the Sc9c130 region of the B chromosome, confirming a terminal deletion. Additionally, 3-D8 showed increased read coverage in the 380–421 Mb region of the B chromosome, suggesting a segmental duplication (Figure 1B and Supplemental Figure 2). Zooming into the terminal region revealed that plant 1-A10 retained most E3900 repeat copies, whereas 3-D8 showed a substantial loss of E3900.

FISH analysis of 1-A10 and 3-D8, together with plants carrying an intact B chromosome, using the B-specific probes D1100, E3900, and Sc9c130, corroborated these findings (Figure 1C and Supplemental Figure 3). In the standard B chromosome, D1100 occupies nearly the entire terminal region of the long arm, except for a small segment. E3900 partially overlaps with D1100 near the subtelomere, whereas Sc9c130 is located at the chromosome end (Figures 1A and 1C). CRISPR–Cas9-mediated cleavage of E3900 in 1-A10 and 3-D8 resulted in the loss of the subtelomeric Sc9c130 FISH signal (Figure 1C and Supplemental Figure 3). Interestingly, 3-D8 showed an enhanced D1100 signal compared with the standard B chromosome (Figure 1C and Supplemental Figure 3), indicating a duplication involving the D1100 array. This pattern may arise from extension of a distal break end over a proximal break end before ligation, followed by deletion at a more distal breakpoint. Alternatively, a breakage–fusion–bridge cycle, which involves the fusion of proximal break ends between sister chromatids followed by random mitotic breakage of the resulting dicentric chromosome in several subsequent mitotic cycles, could be responsible for the observed segmental duplication in the truncated chromosome. This is supported by the occasional observation of a mitotic anaphase bridge involving the B chromosome in plant 3-D8 (Figure 1D). Thus, CRISPR–Cas9-mediated cleavage of rye B-specific satellite repeats enables downsizing of B chromosomes.

To test whether the same approach could be used to reduce the copy number of a standard chromosomal satellite repeat, we targeted the rye–wheat Robertsonian translocation chromosome 1RS/1BL. The Cas9-expressing wheat line (Wang et al., 2022) carries this translocation chromosome, with the rye-specific subtelomeric pSc200 satellite located on its short arm (Figure 1E). A pSc200-specific sgRNA was introduced into five Cas9-positive plants via BSMV (Supplemental Table 1). Seventeen of 18 plants exhibited a substantial reduction in pSc200 FISH signals (Supplemental Tables 3 and 4 and Figure 1F). Nine of 18 plants showed loss of pSc200 signals on at least one 1RS/1BL chromosome, and three plants (17%)

This is an open access article under the CC BY license (http://creativecommons.org/licenses/by/4.0/).

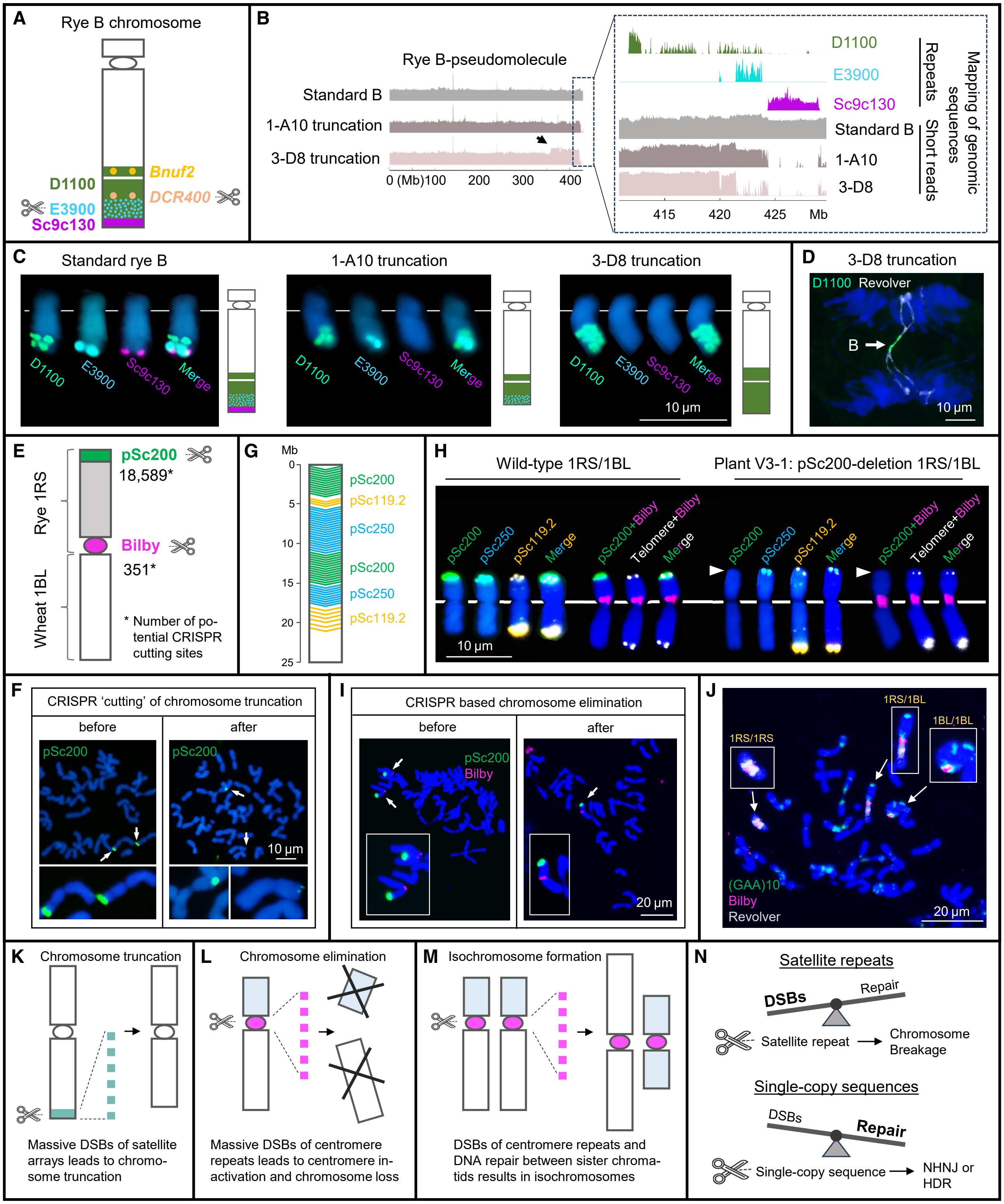

Figure 1. CRISPR–Cas9-mediated truncation and elimination of chromosomes.

(A) Schematic showing the chromosomal distribution of the B chromosome-specific repeats Sc9c130, E3900, and D1100. *Bnuf2* and *DCR400* are B chromosome-specific single-copy genes. Scissors indicate CRISPR-based cutting targets.

(B) Comparative short-read sequencing mapping of plants carrying standard B chromosomes and the B truncation lines (1-A10 and 3-D8) against the complete rye B pseudomolecule. Short-read data from plants carrying two standard B chromosomes and from the B truncation lines 1-A10 and 3-D8

(legend continued on next page)

displayed complete deletion of pSc200 on both 1RS/1BL chromosomes (Supplemental Tables 3 and 4). In the assembled genome of the 1RS/1BL-carrying wheat line Zhou8425B (Li et al., 2025), we identified 18 589 target sites with 100% identity to the pSc200 sgRNA within a 15 Mb subtelomeric region (Figure 1E and Supplemental Table 6).

The 1RS arm features a distal pSc200 array, followed by sequential pSc119.2, pSc250, pSc200, and pSc250 arrays, with a final pSc119.2 array located toward the centromere (Figure 1G). To determine whether CRISPR-mediated deletion of pSc200 also deleted the adjacent pSc250 and pSc119.2 arrays, we performed FISH using pSc250- and pSc119.2-specific probes on plant V3-1, which is homozygous for the pSc200 deletion, as well as a wild-type control (Figure 1H). Despite the absence of pSc200 in V3-1, both pSc250 and pSc119.2 repeats remained at the distal end of chromosome 1RS (Figure 1H and Supplemental Figure 4A), indicating that adjacent repeat arrays toward the centromere were unaffected. Compared with the parental wild-type plant, which shows two adjacent pSc119.2 signal clusters in the subtelomeric region of 1RS, the pSc200-deletion plant retained only one (Figures 1H and Supplemental Figure 4A).

To assess whether the induced chromosome truncations were sealed by *de novo* telomere addition, we analyzed the V3-1 M_1 $_{\text{plant}}$, which exhibited complete deletion of pSc200 signals on both 1RS arms. Telomere-FISH revealed terminal signals on 1RS. Although the wild-type 1RS/1BL chromosome showed stronger telomere signals, the broken chromosome ends of 1RS were sealed with telomeres (Figure 1H and Supplemental Figure 4B).

Next, we tested whether this approach could be used to eliminate an entire chromosome. We focused on the 1RS/1BL chromosome, which carries the rye-specific centromeric repeat *Bilby* that interacts with centromeric histone H3 (CENH3), thereby indicating that the rye-derived centromere of 1RS/1BL is active (Houben et al., 2011; Karimi-Ashtiyani et al., 2021). An sgRNA (Supplemental Table 1) recognizing 351 target sites with 100% identity within the Bilby array (Figure 1E and Supplemental Table 6) was delivered via BSMV into five plants. To detect loss of 1RS/1BL, 16 M_1 plants were analyzed by FISH using the rye probes pSc200 and Bilby. Among these, four (25%) plants were identified as monosomic for 1RS/1BL, indicating loss of one copy of the chromosome (Figure 1I and Supplemental Tables 3 and 5). This result demonstrates that targeted chromosome elimination can be achieved through CRISPR–Cas9-mediated cleavage of centromeric DNA. In addition, plant 0311 carried monosomic 1RS/1BL, as well as 1RS/1RS and 1BL/1BL chromosomes, suggesting that induced double-strand breaks followed by erroneous DNA repair between sister chromatids can generate short- and long-arm monocentric isochromosomes (Figure 1J). In this case, cleavage of centromeric repeats increased the number of chromosomes. To evaluate the specificity of this approach, we performed FISH karyotyping on two selfed progenies derived from wheat monosomic 1RS/1BL ($2n = 41$) plants 032 and 034 (Supplemental Table 5). Both analysed plants contained all wheat chromosomes ($2n = 41$) except one copy of 1RS/1BL (Supplemental Figure 6).

were mapped onto the rye B pseudomolecule. An enlarged view shows read mapping and the distribution of D1100 (green), E3900 (blue), and Sc9c130 (violet) repeats in the terminal 400–429 Mb region of the rye B pseudomolecule.

(C) The standard rye B chromosome and truncated B chromosomes from plants 1-A10 and 3-D8 were analyzed by FISH using the B chromosome-specific repeats D1100 (green), E3900 (blue), and Sc9c130 (violet). Corresponding complete mitotic cells after FISH are shown in Supplemental Figure 3.

(D) Mitotic anaphase of plant 3-D8 showing a B chromosome bridge. FISH was performed using the B chromosome-specific repeat D1100 (green) and the rye genome-specific repeat Revolver (white).

(E) Schematic representation of intermingled pSc200, pSc250, and pSc119.2 repeat arrays within the subtelomeric region (0–25 Mb) of the assembled 1RS chromosome arm in the wheat line Zhou8425B (Li et al., 2025).

(F) CRISPR–Cas9-mediated cleavage of the rye-specific satellite pSc200 (green) resulted in reduced pSc200 FISH signals on one 1RS/1BL chromosome in plant V2-4 (right). The left panel shows the parental wild-type chromosomes after FISH. Arrows indicate typical wild-type pSc200 signals, and the triangle indicates reduced pSc200 FISH signals.

(G) Schematic representation of intermingled pSc200, pSc250, and pSc119.2 repeat arrays within the subtelomeric region (0–25 Mb) of the assembled 1RS chromosome arm in the wheat line Zhou8425B.

(H) Chromosome 1RS/1BL of plant V3-1 shows complete loss of pSc200 FISH signals, whereas telomere, pSc250 and pSc119.2 signals are retained. For comparison, wild-type 1RS/1BL chromosomes after FISH are shown using probes specific for pSc200 (green), *Arabidopsis*-type telomere (white), Bilby (magenta), pSc250 (blue), and pSc119.2 (orange). Corresponding complete mitotic cells after FISH are shown in Supplemental Figure 4. White triangles mark deletion of the pSc200 region.

(I) CRISPR–Cas9-mediated cleavage of the rye centromeric repeat Bilby eliminated one 1RS/1BL chromosome in plant 032 (right). The left panel shows the parental plant of 032 before cutting. FISH was performed using pSc200 (green) and Bilby (magenta). Corresponding interphase nuclei are shown in Supplemental Figure 5.

(J) Plant 0311 possesses a monosomic 1RS/1BL, a monosomic 1RS/1RS isochromosome, and a monosomic 1BL/1BL isochromosome. FISH was performed using the centromeric probes $(GAA)_{10}$ (green) and Bilby (magenta), as well as the rye genome-specific probe Revolver (white). In all FISH images, chromosomes are counterstained with DAPI (blue).

(K–N) Summary of outcomes of CRISPR–Cas9-induced cleavage of satellite DNA.

(K) CRISPR–Cas9-induced cleavage of satellite DNA leads to chromosome truncation. DSBs, double-strand breaks.

(L) CRISPR–Cas9-induced cleavage of a CENH3-associated centromeric repeat results in centromere loss and chromosome elimination.

(M) Alternatively, DNA DSBs in centromeric repeats, followed by erroneous DNA repair between sister chromatids, result in monocentric isochromosomes.

(N) Outcomes differ when Cas9 targets satellite repeats vs. single-copy sequences. When CRISPR targets a satellite repeat, it generates numerous DSBs, overwhelming the cell's DNA repair capacity and leading to chromosome breakage. In contrast, targeting a single-copy sequence produces a single DSB that can be efficiently repaired via non-homologous end joining (NHEJ) or homology-directed repair (HDR).

To investigate whether the same strategy could be used to eliminatenative wheat chromosomes, we targeted the wheat centromeric satellite CentT566 (Su et al., 2019). *In silico* analysis predicted that the CentT566-gRNA selectively targets only four chromosomes—1RS/1BL, 5B, 6B, and 7B—with over 20 perfect matches each (Supplemental Table 6). The centromeric 220–225 Mb region on chromosome 5B harbored the highest density of targets (218 sites; Supplemental Figure 7A and Supplemental Table 6), whereas the centromeres of 1RS/1BL, 6B, and 7B each contained approximately 80–98 target sites (Supplemental Figure 7A and Supplemental Table 6).

CentT566-gRNA was delivered into five plants of the Cas9-positive wheat line via VIGE. Thirty M_1 plants were analyzed by FISH karyotyping to detect chromosome rearrangements. M_1 plant 2-20 exhibited a monosomic chromosome 5B, a monosomic 5BL/5BL isochromosome, and a monosomic telocentric 5BS chromosome (Supplemental Figure 7B). To further evaluate this phenomenon, we karyotyped 17 metaphase cells from plant 2-20. Among these, only six retained 5BS, whereas 14 contained the 5BL/5BL isochromosome and 15 carried chromosome 5B. The lower frequency of 5BS cannot be explained by loss during chromosome preparation; instead, mitotic instability is the likely cause. The presence of a 5BL/5BL isochromosome and a telocentric 5BS strongly suggests that CRISPR–Cas9 induced chromosome rearrangements at the centromere of chromosome 5B.

In conclusion, VIGE-mediated CRISPR targeting of satellite repeats expands the chromosome engineering toolkit. The high efficiency likely arises from numerous double-strand breaks that overwhelm the repair machinery, leading to truncation, loss, or isochromosome formation (Figures 1K–1N). Consistent with this interpretation, a very low frequency of chromosomal changes has been reported when using a single-copy gRNA target (Samach et al., 2023). Although limited by lower specificity and repeat-dependent cleavage sites, satellite-specific gRNAs enable targeted chromosome truncation and elimination. This strategy facilitates the transfer of chromosomal segments from wild relatives into wheat, enhances genetic diversity and breeding potential, and enables the generation of minichromosomes as future engineered genetic platforms (Supplemental Note 2).

DATA AND CODE AVAILABILITY

The short-read sequencing data for plant 1-A10 (run accession number: ERR14952793) and 3-D8 (run accession number: ERR14952833) are available in the European Nucleotide Archive (ENA) under project PRJEB89395. Data for wheat with standard B chromosomes (Chen et al., 2024) are available under accession PRJEB69479 (run accession number: ERR12870557).

FUNDING

This work was supported by the German Research Foundation (projects 559365585 and 395162636 to A.H.) and the China Scholarship Council (CSC202006850005 to J.C.).

ACKNOWLEDGMENTS

We gratefully acknowledge T. Endo (Japan) for providing valuable wheat seeds carrying the rye B chromosome and E. Akhunov (USA) for supplying the high-Cas9 wheat line 707. We thank I. Schubert (IPK, Germany) for fruitful discussions. We also thank O. Weiss, K. Kumke, and J. Lorenz for excellent technical assistance; A. Fiebig and M. Maruschewski (IPK, Germany) for support with sequence submission; and J. Fuchs for assistance with figure preparation. No conflict of interest declared.

AUTHOR CONTRIBUTIONS

J.C. performed most experiments, including plant cultivation, FISH, VIGE, PCR, and data analysis. T.L., L.B., S.T.-N.-A., and S.H. performed VIGE. Y.X. and Z.Q. performed karyotyping. J.P. and H.G. conducted genotyping. A.H. supervised the research project. J.C. and A.H. wrote the manuscript with input from all coauthors.

SUPPLEMENTAL INFORMATION

Supplemental information is available at *Plant Communications Online*.

Received: January 26, 2026
Revised: February 24, 2026
Accepted: March 20, 2026
Published: March 23, 2026

Jianyong Chen[1,], Taoran Liu[1], Yating Xia[2], Luisa Barth[1], Jörg Plieske[3], Heike Gnad[3], Suriya Tamilselvan-Nattar-Amutha[1], Zengjun Qi[2], Stefan Heckmann[1] and Andreas Houben[1,4,*]*

[1]Leibniz Institute of Plant Genetics and Crop Plant Research (IPK), Gatersleben, 06466 Seeland, Germany
[2]State Key Laboratory of Crop Genetics and Germplasm Enhancement & Utilization, Nanjing Agricultural University, Nanjing 210095, China
[3]SGS INSTITUT FRESENIUS GmbH, TraitGenetics Section, Am Schwabeplan 1b, 06466 Seeland OT Gatersleben, Germany
[4]Martin Luther University Halle-Wittenberg, Institute of Agricultural & Nutritional Sciences, Halle, Saale, Germany
***Correspondence: Jianyong Chen (chenj@ipk-gatersleben.de), Andreas Houben (houben@ipk-gatersleben.de)**
https://doi.org/10.1016/j.xplc.2026.101833

REFERENCES

Chen, J., Bartoš, J., Boudichevskaia, A., Voigt, A., Rabanus-Wallace, M.T., Dreissig, S., Tulpová, Z., Šimková, H., Macas, J., Kim, G., et al. (2024). The genetic mechanism of B chromosome drive in rye illuminated by chromosome-scale assembly. Nat. Commun. **15**:9686.

Endo, T.R., Nasuda, S., Jones, N., Dou, Q., Akahori, A., Wakimoto, M., Tanaka, H., Niwa, K., and Tsujimoto, H. (2008). Dissection of rye B chromosomes, and nondisjunction properties of the dissected segments in a common wheat background. Genes Genet. Syst. **83**:23–30.

Houben, A., Kumke, K., Nagaki, K., and Hause, G. (2011). CENH3 distribution and differential chromatin modifications during pollen development in rye (*Secale cereale* L.). Chromosome Res. **19**:471–480. https://doi.org/10.1007/s10577-011-9207-6.

Karimi-Ashtiyani, R., Schubert, V., and Houben, A. (2021). Only the rye derived part of the 1BL/1RS hybrid centromere incorporates CENH3 of wheat. Front. Plant Sci. **12**:802222.

Li, G., Ren, Y., Yang, Y., Chen, S., Zheng, J., Zhang, X., Li, J., Chen, M., Sun, X., Lv, C., et al. (2025). Genomic analysis of Zhou8425B, a key founder parent, reveals its genetic contributions to elite agronomic traits in wheat breeding. Plant Commun. **6**:101222.

Samach, A., Mafessoni, F., Gross, O., Melamed-Bessudo, C., Filler-Hayut, S., Dahan-Meir, T., Amsellem, Z., Pawlowski, W.P., and Levy, A.A. (2023). CRISPR/Cas9-induced DNA breaks trigger crossover, chromosomal loss, and chromothripsis-like rearrangements. Plant Cell **35**:3957–3972. https://doi.org/10.1093/plcell/koad209.

Su, H., Liu, Y., Liu, C., Shi, Q., Huang, Y., and Han, F. (2019). Centromere satellite repeats have undergone rapid changes in polyploid wheat subgenomes. Plant Cell **31**:2035–2051. https://doi.org/10.1105/tpc.19.00133.

Tamilselvan-Nattar-Amutha, S., Hiekel, S., Hartmann, F., et al. (2023). Barley stripe mosaic virus-mediated somatic and heritable gene editing in barley (*Hordeum vulgare* L.). Front. Plant Sci. **14**:1201446.

Wang, W., Yu, Z., He, F., Bai, G., Trick, H.N., Akhunova, A., and Akhunov, E. (2022). Multiplexed promoter and gene editing in wheat using a virus-based guide RNA delivery system. Plant Biotechnol. J. **20**:2332–2341.

Yaşar, S., Gehrke, F., Capdeville, N., and Puchta, H. (2026). Recent progress in plant genome engineering: from large insertions to chromosome number changes. Curr. Opin. Biotechnol. **97**:103426. https://doi.org/10.1016/j.copbio.2025.103426.

Plant Communications, Volume 7

Supplemental information

Satellite DNA-targeted CRISPR–Cas9-mediated editing enables chromosome truncation and elimination in wheat

Jianyong Chen, Taoran Liu, Yating Xia, Luisa Barth, Jörg Plieske, Heike Gnad, Suriya Tamilselvan-Nattar-Amutha, Zengjun Qi, Stefan Heckmann, and Andreas Houben

Supplementary information

Satellite DNA-targeted CRISPR/Cas9-mediated editing enables chromosome truncation and elimination in wheat

Jianyong Chen[1*], Taoran Liu[1], Yating Xia[2], Luisa Barth[1], Jörg Plieske[3], Heike Gnad[3], Suriya Tamilselvan-Nattar-Amutha[1], Zengjun Qi[2], Stefan Heckmann[1] & Andreas Houben[1,4*]

[1] Leibniz Institute of Plant Genetics and Crop Plant Research (IPK), Gatersleben, 06466 Seeland, Germany

[2] State Key Laboratory of Crop Genetics and Germplasm Enhancement & Utilization, Nanjing Agricultural University, 210095 Nanjing, China

[3] SGS INSTITUT FRESENIUS GmbH, TraitGenetics Section, Am Schwabeplan 1b, 06466 Seeland OT Gatersleben, Germany

[4] Martin Luther University Halle Wittenberg, Institute of Agricultural & Nutritional Sciences, Halle, Saale, Germany

* Corresponding Authors:

Andreas Houben, e-mail: houben@ipk-gatersleben.de

Jianyong Chen, e-mail: chenj@ipk-gatersleben.de

Supplementary Note 1

Plant chromosome engineering, aimed at enhancing genetic variation, began with the pioneering work of (Sears 1956), who used X-ray irradiation to induce chromosome translocations between wheat and its wild relative, *Aegilops umbellulata.* Other methods to change the combination of genes or structure of plant chromosomes included

heterozygous combination of already rearranged chromosome complements (Schubert and Rieger 1985; Schubert and Oud 1997), the application of the gametocidal system (Shi and Endo 1997), the breakage-fusion-bridge cycle (Lukaszewski 1997) or the Pairing Homoeologous (Ph) system (Riley and Chapman 1958; Sears and Okamoto 1958).

With the development of CRISPR/Cas9 systems, the targeted depletion of chromosomes in mammalian cell cultures has become feasible through the application of single or multiplexed guide RNAs (Zuo et al. 2017; Adikusuma et al. 2017; Hashizume et al. 2025). However, the elimination of an entire plant chromosome by this technology has not yet been demonstrated. So far, CRISPR/Cas9 targeting centromeric and rDNA tandem repeats has been used to induce cell death in specific organs and at particular time points in a controlled manner in *A. thaliana* (Schindele et al. 2022; Gehrke et al. 2023). Chromosome rearrangements using guide RNA (gRNA) targeting a unique genomic sequence are now possible in plants, as demonstrated in *Arabidopsis thaliana* (Schmidt et al. 2020; Schmidt et al. 2019; Ronspies et al. 2025), maize (Schwartz et al. 2020), and wheat (Zhao et al. 2025).

Notable, terminal chromosome truncation, chromosome loss and segmental duplications as well as evidence of bridges that could explain some of the observed phenomena following DSB induction. This was obtained at low frequency but using a single copy gRNA target, suggesting that defective repair is not exclusively taking place when repetitive arrays are targeted (Samach et al. 2023).

Another goal in plant chromosome engineering is the generation of minichromosomes, which could be used to stack genes of interest from any source, potentially serving as the first step toward synthesizing a complete engineered plant genome. A 'top-down' approach is preferable to *de novo* minichromosome synthesis, as it begins with the native chromosomes and shortens chromosome arms as much as possible to minimize dosage effects and to achieve phenotypic neutrality (reviewed by (Birchler et al. 2024)). A pioneering study used the *Agrobacterium*-mediated insertion of the telomere repeat to

induce chromosomal truncation in maize (Yu et al. 2006). Later, this method was applied to *Arabidopsis* (Nelson et al. 2011; Teo et al. 2011), barley (Kapusi et al. 2012), rice (Xu et al. 2012), wheat (Yuan et al. 2017), and *Brassica* (Yan et al. 2017). The drawback of the hitherto performed plant chromosome-truncation experiments is the largely random downsizing of chromosomes. For a recent review of plant genome editing see (Puchta and Houben 2024).

Supplementary Note 2

Although heterochromatic features typically lower the CRISPR/Cas9 mutagenesis efficiency for single-copy sequences, as observed across multiple species, including plants (Weiss et al. 2022; Daer et al. 2017), centromeric and noncentromeric satellite DNA arrays proved suitable for CRISPR/Cas9-mediated chromosome engineering. This success aligns with the established use of Cas9 for the *in situ* detection of various satellite repeats, which has been demonstrated in living (Dreissig et al. 2017; Anton et al. 2014), as well as in fixed cells, across species (Ishii et al. 2019; Potlapalli et al. 2025; Potlapalli et al. 2024; Potlapalli et al. 2020; Deng et al. 2015). Thus, satellite repeats are effective targets for CRISPR/Cas-based chromosome engineering, despite their potentially less favourable heterochromatic chromatin features. Most likely, the high copy number compensates for local inhibition of CRISPR/Cas9-based DNA fragmentation.

Unlike CRISPR-induced cutting of 45S rDNA and 178-bp centromere repeats in diploid *A. thaliana*, which triggers cell death (Schindele et al. 2022; Gehrke et al. 2023), polyploid wheat and B chromosomes tolerate extensive DNA loss. This combination provides a powerful platform for developing and testing chromosome-engineering approaches in complex plant genomes. This strategy can be applied even to generate minichromosomes via a ‘top-down’ approach. Unlike the *Agrobacterium*-mediated telomere insertion for achieving random chromosome truncation (Yu et al. 2007; Yu et al. 2006), our approach enables the precise targeting of specific chromosomes by cleaving defined

pericentromeric or other satellite repeats, thereby facilitating the engineering of size-reduced chromosomes as a future shuttle system.

Furthermore, targeted chromosome elimination can accelerate breeding efforts in polyploid crops. For example, a wheat-rye 6R addition line could be crossed with a 6D nullisomic wheat line to produce a double monosomic 6R/6D line, allowing the wheat and rye homeologs to pair and recombine during meiosis in the *ph* mutant background. However, complete wheat hypoploid stocks (nullisomics, monosomics) are limited to the genotype 'Chinese Spring' (Endo and Gill 1996; Sears 1954). Therefore, developing a CRISPR-based targeted chromosome elimination in diverse wheat genotypes could facilitate subsequent alien chromosome introgression using the *Ph* system. Whether the elimination of an entire paternal chromosome set by CRISPR-mediated centromere-repeat targeting can effectively serve as a method for generating haploids remains to be tested. For this aim, the centromere repeat composition must be either distinct between the haploid inducer expressing the gRNA/CRISPR construct and the target genotype, or the CRISPR-triggered chromosome elimination must be restricted to the male or female gametes of the haploid inducer.

The VIGE system represents a highly efficient platform for chromosome engineering in crops. Once a Cas9 transgenic plant line has been established, different repeats can be readily targeted by modifying the BSMV vectors carrying the corresponding sgRNAs through simple cloning steps. This strategy enables rapid, flexible genome editing by bypassing the lengthy transformation steps typically required, as shown in our study. In the future, more efficient virus-based approaches can further improve the efficiency of the CRISPR/Cas system. The application of an intronized Cas9 gene might be one way, because the incorporation of introns into the coding sequence strongly enhanced the editing performance in dicots (Grützner et al, 2021) and monocots (Lawrenson et al, 2024). or complemented to enable controlled editing of single-copy loci. Application of the highly efficient Cas enzyme might enable controlled editing of single-copy loci and target repeats for simultaneous chromosome and gene engineering in the future.

Materials and methods

Plant material

Wheat (*Triticum aestivum* L., cv. 'Chinese Spring') with two standard rye B chromosomes (Endo et al. 2008) and transgenic wheat cv. 'Bobwhite' with high levels of Cas9 expression (high-Cas9 line 707) (Wang et al. 2022) were grown in glasshouses (Gatersleben, Germany). Wheat cv. 'Chinese Spring' with two rye B chromosomes was pollinated by the high-Cas9 line 707 to create Cas9-expressing wheat with rye B chromosomes. Cas9-positive plants were selected by PCR using the primer pair zCas9-F and zCas9seq1(Supplementary Table 1) (Wang et al. 2022).

Genomic DNA isolation

Leaf segments (1 - 3 cm) from rye and wheat were frozen in tubes by immersion in liquid nitrogen and pulverized by bead beating in a vibrating mill (MM400, Retsch, frequency: 30/s, 1 min). Extraction buffer (1.21 g Tris, 4.09 g NaCl, 1.86 g EDTA, diluted in 100 ml H_20; 1.2 ml/sample) was added to each tube and incubated for 15 min at 65 °C. Tubes were allowed to cool for 1 min at room temperature (RT). Chloroform: isoamyl alcohol (24:1, 600 µl) was added, and the solution was manually agitated. The homogenate debris was pelleted by centrifugation (13,000 g for 2 min), and the supernatant containing DNA was transferred to a fresh tube. The DNA was precipitated in 700 µl isopropanol, pelleted, and washed twice in 70% ethanol by repeated vortexing and centrifugation (13,000 g for 2 min). The pellet was air-dried until all ethanol was evaporated, and the DNA was dissolved in 50 µl of dH20. The DNA concentration was determined by spectrophotometry (Thermo Scientific NanoDrop One) and the Qubit device (DNA HS assay kit, Thermo Fisher Scientific Inc, Waltham, MA, USA).

Preparation of chromosome spreads

To prepare mitotic chromosomes, seeds were germinated on wet filter paper at room temperature (RT) for 2-3 days. Excised roots were treated with ice-cold water for 24 h for

cell cycle synchronization, fixed in 3:1 ethanol: acetic acid for 1-3 days at RT, and stored in 70% ethanol at -20 °C. To prepare meiotic chromosomes, young spikes were collected when the 1/3 of the flag leaves were out and fixed in 3:1 ethanol: acetic acid for 3 days at RT. Roots or young anthers were treated with 45% acetic acid between 10 and 120 min before being transferred onto a glass slide. Meristematic cells were then isolated in a droplet of 45% acetic acid under a coverslip. After 2 sec of heating over an ethanol burner, the meristem was squashed between the slide and coverslip. Finally, the coverslip was removed after freezing it in liquid nitrogen, and the slide was kept in 99.8% ethanol at -20 °C until use.

Fluorescence in situ hybridization (FISH), probe generation and microscopy

The PCR products and plasmids used as FISH probes were fluorescence-labelled by nick translation (NT Labeling Kit, Jena Bioscience). The repeat Sc9c130 was obtained by PCR. The following plamids were used: pTZE3900, containing a 3.9 kb fragment of the repeat E3900 (Blunden et al. 1993), pUC119-Revolver, containing a 89 bp sequence of the rye-genome specific repeat Revolver (Tomita et al. 2008), pUC18-pSc200 containing a 521 bp fragment of the repeat pSc200 (Vershinin et al. 1995), and a 3.4 kb fragment of the rye-specific centromeric repeat Bilby (Francki 2001). The following oligonucleotide (oligo) probes, each labeled at the 5′ end with a fluorophore, were used in this study: four oligos (30 nt - 40 nt, Oligo-D1100-mix) with fluorescein isothiocyanate (FITC) targeted the sequence of the repeat D1100 (Sandery et al. 1990a, b); Oligo-pSc200-6 targeting the pSc200 repeat (Guo et al. 2019), labeled with 6-carboxyfluorescein (FAM); Oligo-pSc250 targeting the pSc250 repeat (Fu et al. 2015), labeled with 5(6)-carboxytetramethylrhodamine (TAMRA); Oligo-pSc119.2-1 targeting the pSc119.2 repeat and microsatellite $(GAA)_{10}$ (Huang et al. 2018), labeled with FAM; and an oligo probe targeting the *Arabidopsis*-type telomere sequence, also labeled with TAMRA. The sequences of primers and oligo probes are listed in Supplementary Table 1.

For FISH, chromosomes were denatured in a NaOH - 70% ethanol solution (6 mg/ml) for 5 min at RT, then washed, dehydrated in a series of increasing ethanol concentrations

(70%, 90%, and 99.8%) for 5 min each, and air-dried. For each FISH probe, 1 µl of probe (10 mM for oligo probes;50-75 ng/µl for probes generated by nick translation) was added to 10 µl hybridization mixture (Aliyeva-Schnorr et al. 2015), denatured at 99 °C for 10 min and stored immediately at -20 °C until use. The mixture was added to the air-dried slides, sealed with coverslips, and incubated in a moist chamber at 37 °C for 12-24 h. After hybridization, slides were washed in 2x SSC for 20 min at 58 °C and distilled water at RT for 2 min, and air dried. Finally, 8 µl 4',6-diamidino-2-phenylindole (DAPI) solution (1 µg/mL, DAPI/antifade solution) in antifade was added to each slide and sealed with a coverslip. Microscope images were taken using an Olympus BX61 fluorescence microscope equipped with an ORCA-ER CCD camera (Hamamatsu). Images were analyzed using the cellSens Dimension software (Olympus, v1.11) and Adobe Photoshop (v13.0).

Karyotyping of wheat chromosomes

For wheat karyotyping, slide preparation followed a modified protocol in which root tips were treated as described by (Huang et al. 2018) to accumulate metaphase chromosomes. Next, root meristems were transferred into a 0.5 mL microcentrifuge tube containing enzymatic digestion solution (for 10 ml solution, 0.3 g cellulase, 0.1 g pectinases, 2 ml 5× citrate buffer (pH 5.5), and 8 ml ddH_2O) and digested at 37 °C for 35 - 40 min. Then, meristems were washed twice with distilled water and twice with absolute ethanol (5 min each wash). 10 ml of 100% acetic acid was added to each tube, and the root tips were macerated using a homogenizer until a uniform cell suspension was obtained. Microscope slides were placed in a humid chamber, and the cell suspension was dropped onto the slide surface. Slides were incubated in the chamber for 30 min to facilitate chromosome spreading. For FISH, the same procedure was used, and the multiplex oligonucleotide probe ONPM#7 (Chen et al. 2019) (Supplementary Table 1), which contains different 13 oligos that distinguish all wheat chromosomes, was applied.

sgRNA design, cloning and in silico analysis

Following the design guidelines (Schindele et al. 2020), a single sgRNA was designed for each of the following sequences (Supplementary Table 1): the E3900 repeat (GenBank: AF222021.1), the pSc200 repeat (GenBank: Z50039.1), the Bilby repeat (GenBank: AF245032.1), and the single-copy B chromosome-specific gene *DCR400* (Chen et al. 2024). Each sgRNA was subsequently cloned into the generic guide RNA expression vector BSMV-γ-sg, as described (Tamilselvan-Nattar-Amutha et al. 2023). The sgRNAs of pSc200, and Bilby were aligned to the 1RS/1BL reference sequence of the wheat line Zhou8425B (Li et al., 2025) using BLASTn (E-value = 10, maximum target sequences = 10,000) through WheatOmics (Ma et al. 2021) to *in silico* determine their target number. Only alignments with 100% identity over 20 base pairs were considered.

In vitro transcription of the virus and plant inoculation

The *in vitro* transcription of Barley stripe mosaic virus (BSMV) was as described (Wang et al. 2022) with modifications. ~3 - 4 kb DNA fragments of BSMV α, β and γ were amplified using T7 primers (Supplementary Table 1) and ExTaq polymerase (Takara Bio). The PCR products were purified using the Monarch PCR & DNA Cleanup Kit (NEB) and transcribed *in vitro* to α, β and γ virus chains using HiScribe T7 High Yield RNA synthesis kit (New England BioLabs, catalog number E2050S) following the Capped RNA Synthesis protocol provided by the manufacturer. The m7G(5′)ppp(5′)G RNA Cap (New England BioLabs, catalog number S1404L) was used as Cap Analog with 4:1 of Cap Analog: GTP ratio. The quality and concentration of RNA transcripts (usually 2–2.5 µg/µl) were assessed on the agarose gel.

The second leaf of wheat seedlings at the two-leaf stage was inoculated using the mixture of 60 µl phosphate buffer and transcription products of BSMV α, β, and γ chains (2.5 µl each). The phosphate buffer (10 mM) (pH=7.0) contains 0.5% each of celite 545 (Roth) and silicon carbide (400 mesh particle size, Sigma-Aldrich). The inoculation was performed by hand-rubbing the second leaf from the base to the tip while wearing clean nitrile gloves. The procedure was repeated three times for each plant, each time applying

20 µl of the mixture. After inoculation, the plants were kept in the dark overnight. The virus infection symptoms were usually observed 7-10 days after inoculation. The work was performed in a biosafety level 2 (S2) laboratory. The plants used for sequencing and other analyzis were checked by Reverse Transcription-PCR (RT-PCR) using the primer pair gammaF+ gammaR (Supplementary Table 1) to ensure the absence of virus.

Genotyping the chromosomal deletion on the rye B chromosome

After harvesting the seeds from the infected plants, a pair of primers Bnuf2-1 (Supplementary Table 1) targeting the kinetochore nuf2 gene on the B chromosome (Chen et al. 2024) was used to confirm the presence of the rye B chromosome in the progenies. Sc9c130-specific primers were used to confirm the presence of the chromosome end of the rye B chromosome in the progenies (Supplementary Table 1). PCR genotyping was performed using GoTaq DNA Polymerase (Promega) according to the manufacturer's recommended protocol. PCR products were analyzed on 1% agarose gels. For the single-copy gene Bnuf2-1, PCR amplification was performed with 35 cycles; 25 cycles were used for the high-copy repeat Sc9c130.

Short-read sequencing and mapping

For short-read sequencing, the genomic DNA of plants 1-A10 and 3-D8 carrying a rye B chromosome truncation was sequenced using the DNBSEQ-T7 sequencing platform at BGI Genomics (Hong Kong, China). About 35 Gb paired-end 150 (PE150) data were generated for each sample. Both datasets were aligned to the wheat genome (IWGSC RefSeq v2.1 assembly) plus rye B-pseudomolecule using bowtie2 (v2.5.0, default) (Langmead and Salzberg 2012). The alignments on the B-pseudomolecule and unassigned rye B-like contigs were extracted via samtools (v1.9) (Li et al. 2009) and visualized by pyGenomeTracks (v3.8) (Lopez-Delisle et al. 2021).

Supplementary Table 1

Oligos used in this study

ID	Category	SEQUENCE (5'-3')
Oligo-D1100-1	FISH probe	5'- FITC-ACCGCATCTCCCTCACTCACAATTTCGATTCCTCCTT-3′
Oligo-D1100-2	FISH probe	5'- FITC-GGTCTCGTTTCCCGCCCAAAGTTTCGCCCC-3′
Oligo-D1100-3	FISH probe	5'- FITC-GTATAGCAAAAGAGTTTCCCAAATAGGCGGCACGA-3′
Oligo-D1100-4	FISH probe	5'- FITC-CGGGTATGGGAACGTAGCATGGAGTTTGGTGG-3′
Arabidopsis-type telomere	FISH probe	5'-TAMRA-GGGTTTAGGGTTTAGGGTTT-3′
Oligo-pSc200-6	FISH probe	5'-FAM-CCATACATGGGTGGGGCGCCAAGGACCTGAACACCAAAGTGATATGCCGGCTCATCAAA-3′
Oligo-pSc250	FISH probe	5'-TAMRA-TGTGTTGTTCTTGGACAAAACAATGCATACCATCTCTTCTAC-3'
GAA10	FISH probe/ONPM#7	5′-FAM-GAAGAAGAAGAAGAAGAAGAAGAAGAAGAA-3'
Oligo-pSc119.2-1	FISH probe/ONPM#7	5′-TAMRA (or FAM)-GGCCAGAATCGGCCAAAACTACGAGTGCTGATGACCGACACGTAAACGCACCCCGGGGT-3′
Oligo-pAs1-1	FISH probe/ONPM#7	5′-TAMRA-GGATGCACTTCGTGTACAAAACGGACAATCTCTTTCAAAGTATCAGGATTTCATCC-3′
Oligo-pAs1-3	FISH probe/ONPM#7	5′-TAMRA-TTTTTGTGTGTTCAAAATGCACCATTCAAAGCCACATCATCATTTTTCAATCCTTT-3′
Oligo-pAs1-4	FISH probe/ONPM#7	5′-TAMRA-CTGACTTCATTTGTTATTTTTCATGCATTTACTAATTATTTTTAGCTATAAGACCC-3′
Oligo-pAs1-6	FISH probe/ONPM#7	5′-TAMRA-CATCATTTCATCCACATAGCATGTGCAAGAAAGTTGAGAGGGTTACGGCAAAAACT-3′
Oligo-AFA-3	FISH probe/ONPM#7	5′-TAMRA-AAGTATCAGGGTTTCGGACGGAAACTCATCTATTACAAAGGGATT-3′
Oligo-AFA-4	FISH probe/ONPM#7	5′-TAMRA-CAGTTTTTAAAACATATTTGAACTCCTGACTTTTTGTGTGTT-3′
Oligo-5S-1	FISH probe/ONPM#7	5′-TAMRA-TCATACCAGCACTAAAGCACCGGATCCCATCAGAAC-3′
Oligo-5S-2	FISH probe/ONPM#7	5′-TAMRA-GCGTGCTTGGGCGAGAGTAGTACTAGGATGGGTGAC-3′
Oligo-BSCL135-1	FISH probe/ONPM#7	5′-FAM-GCCTCGACTCGCGTTACCCTAAGATAGACAG-3′
Oligo-BSCL135-2	FISH probe/ONPM#7	5′-FAM-GTGGTGTCACGTGCTCGTCTCGGCGTCCGT-3′
Oligo-BSCL242-1	FISH probe/ONPM#7	5′-FAM-GTTGCTCAAACTAGAAAATGGACAGATATGGT-3′

Continue: Supplementary Table 1 Oligos used in this study

ID	Category	SEQUENCE (5'-3')
NCR400_g1F	sgRNA cloning	GTAAGAAGGAAACCAGGAAGAAGTGTTT
NCR400_g1R	sgRNA cloning	TCTAAAACACTTCTTCCTGGTTTCCTTC
gBilby-2F	sgRNA cloning	GTAAGGGGTGAAGGAGGGTCCATGGTTT
gBilby-2R	sgRNA cloning	TCTAAAACCATGGACCCTCCTTCACCCC
gE3900-6F	sgRNA cloning	GTAACAGGTGATGGATCGAGACAGGTTT
gE3900-6R	sgRNA cloning	TCTAAAACCTGTCTCGATCCATCACCTG
gpSc200-1F	sgRNA cloning	GTAATAGGCGTTGGAAAGCTATTGGTTT
gpSc200-1R	sgRNA cloning	TCTAAAACCAATAGCTTTCCAACGCCTA
gCenTt566-7F	sgRNA cloning	GTAATACTCCCCGCTTGCGCCAGGGTTT
gCenTt566-7R	sgRNA cloning	TCTAAAACCCTGGCGCAAGCGGGGAGTA
zCAS9-F	PCR primer	CGGACTAGTATGGATTACAAGGACCACGACG
zCAS9seq1	PCR primer	ACCTTGTACTCGTCGGTGATCAC
Sc9c130-fwd	PCR primer	GCATGTCATCGGTAGGATAGG
Sc9c130-rev	PCR primer	ACCCCTTCCCTTTCGATCTAC
Bnuf2-1F	PCR primer	TGGTTCCTCTCGCTTGTTCC
Bnuf2-1R	PCR primer	GGCGAGTGTGAAGTCCTTGA
[1]BSMValpha_T7_F	T7 primer	TAATACGACTCACTATAGTATGTAAGTTGCCTTTGGG
[1]BSMVbeta_T7_F	T7 primer	TAATACGACTCACTATAGTAAAAGAAAAGGAACAACC
[1]BSMVgamma_T7_F	T7 primer	TAATACGACTCACTATAGTATAGCTTGAGCATTACCG
[1]BSMVcommon_R	T7 primer	TGGTCTTCCCTTGGGGGACC
gammaF	Virus-detection	TGGCTAAGCTTGAAAGTGAGG
gammaR	Virus-detection	TAAAGTGTGACGCAGCTACC

1: amplification of α, β, and γ are using their corresponding left primers and the common right primer BSMVcommon_R.

Supplementary Table 2

B chromosome truncations detected by PCR

sgRNA target	Screened plants	B-positive plants [a]	Plants with B truncation	Frequency of B truncations [b]
E3900	179	82	8	9.8%
DCR400	182	86	0	0%

a Only a subset of the plants carried the B chromosome, and those selected for VIGE contained only a single rye B chromosome. To identify the presence of the rye B chromosome, we used primers (Bnuf2) targeting the rye B-specific variant of the kinetochore protein nuf2 (321,949,858-321,954,295 bp on the assembled B sequence).

b The frequency of induced truncations = (Plants with B-deletion / B-positive plants)

Supplementary Table 3

Statistics of CRISPR/Cas9-mediated ‘cutting’ of pSc200 and Bilby repeats on 1RS

Number of screened plants for cutting pSc200	Plants with reduced pSc200 signals	Plants with complete loss of the pSc200 FISH signal
18	17 (94.4%)	3 (16.7%)

Number of screened plants for cutting Bilby	Plants with monosomic 1RS/1BL	Plants with monosomic 1RS/1RS
16	4 (25%)	1 (6.3%)

Supplementary Table 4

FISH result of the M_1 plants from CRISPR-based 'cutting of pSc200

Plant name	pSc200 homolog chromosome I	pSc200 homolog chromosome II	Note
Parent	+	+	normal
V1-1	+	-	reduction
V1-2	+	-	reduction
V1-3	+	lost	heterozygous deletion
V1-4	+	-	reduction
V2-1	+	-	reduction
V2-2	+	+	normal
V2-3	+	-	reduction
V2-4	+	-	reduction
V2-5	-	lost	heterozygous deletion
V2-6	lost	lost	homozygous deletion
V2-7	-	lost	heterozygous deletion
V2-8	-	lost	heterozygous deletion
V2-9	+	-	reduction
V3-1	lost	lost	homozygous deletion
V3-2	-	-	reduction
V3-3	lost	lost	homozygous deletion
V3-4	-	lost	heterozygous deletion
V3-5	+	lost	heterozygous deletion

Note: '+' indicates FISH signals similar to the wild-type parent. '-' indicates signals substantially reduced compared to the wild-type parent. 'lost' indicates no pSc200-signal.

Supplementary Table 5

Analysis of the M_1 plants from CRISPR/Cas9-based ‘cutting of the Bilby repeat. Bilby and pSc200s-specific probes were used for FISH.

Plant name	FISH result
Parent	Disomic 1RS/1BL
035	Monosomic 1RS/1BL
032	Monosomic 1RS/1BL
034	Monosomic 1RS/1BL
0314	Monosomic 1RS/1BL
0311	Monosomic 1RS/1BL, monosomic 1RS/1RS, and monosomic 1BL/1BL
143	Disomic 1RS/1BL
031	Disomic 1RS/1BL
033	Disomic 1RS/1BL
141	Disomic 1RS/1BL
142	Disomic 1RS/1BL
041	Disomic 1RS/1BL
042	Disomic 1RS/1BL
044	Disomic 1RS/1BL
045	Disomic 1RS/1BL
0312	Disomic 1RS/1BL
0313	Disomic 1RS/1BL

Supplementary Table 6

Genome-wide prediction of target sites for sgRNAs used in this study

Reference

Adikusuma F, Williams N, Grutzner F, Hughes J, Thomas P (2017) Targeted Deletion of an Entire Chromosome Using CRISPR/Cas9. Molecular therapy : the journal of the American Society of Gene Therapy. doi:10.1016/j.ymthe.2017.05.021

Aliyeva-Schnorr L, Beier S, Karafiátová M, Schmutzer T, Scholz U, Doležel J, Stein N, Houben A (2015) Cytogenetic mapping with centromeric bacterial artificial chromosomes contigs shows that this recombination-poor region comprises more than half of barley chromosome 3 H. The Plant Journal 84 (2):385-394

Anton T, Bultmann S, Leonhardt H, Markaki Y (2014) Visualization of specific DNA sequences in living mouse embryonic stem cells with a programmable fluorescent CRISPR/Cas system. Nucleus 5 (2):163-172. doi:10.4161/nucl.28488

Birchler JA, Kelly J, Singh J, Liu H, Zhang Z, Char SN, Sharma M, Yang H, Albert PS, Yang B (2024) Synthetic minichromosomes in plants: past, present, and promise. The Plant Journal 120 (6):2356-2366

Blunden R, Wilkes TJ, Forster JW, Jimenez MM, Sandery MJ, Karp A, Jones RN (1993) Identification of the E3900 family, a 2nd family of rye chromosome-B specific repeated sequences. Genome / National Research Council Canada = Genome / Conseil national de recherches Canada 36 (4):706-711

Chen J, Bartoš J, Boudichevskaia A, Voigt A, Rabanus-Wallace MT, Dreissig S, Tulpová Z, Šimková H, Macas J, Kim G, ... & Houben, A. (2024) The genetic mechanism of B chromosome drive in rye illuminated by chromosome-scale assembly. Nature Communications 15 (1):9686

Chen J, Tang Y, Yao L, Wu H, Tu X, Zhuang L, Qi Z (2019) Cytological and molecular characterization of Thinopyrum bessarabicum chromosomes and structural rearrangements introgressed in wheat. Molecular Breeding 39 (10):1-14

Daer RM, Cutts JP, Brafman DA, Haynes KA (2017) The Impact of Chromatin Dynamics on Cas9-Mediated Genome Editing in Human Cells. Acs Synth Biol 6 (3):428-438. doi:10.1021/acssynbio.5b00299

Deng WL, Shi XH, Tjian R, Lionnet T, Singer RH (2015) CASFISH: CRISPR/Cas9-mediated in situ labeling of genomic loci in fixed cells. P Natl Acad Sci USA 112 (38):11870-11875. doi:10.1073/pnas.1515692112

Dreissig S, Schiml S, Schindele P, Weiss O, Rutten T, Schubert V, Gladilin E, Mette MF, Puchta H, Houben A (2017) Live-cell CRISPR imaging in plants reveals dynamic telomere movements. Plant J 91 (4):565-573. doi:10.1111/tpj.13601

Endo T, Gill B (1996) The deletion stocks of common wheat. Journal of Heredity 87 (4):295-307

Endo TR, Nasuda S, Jones N, Dou Q, Akahori A, Wakimoto M, Tanaka H, Niwa K, Tsujimoto H (2008) Dissection of rye B chromosomes, and nondisjunction properties of the dissected segments in a common wheat background. Genes & genetic systems 83 (1):23-30

Francki MG (2001) Identification of Bilby, a diverged centromeric Ty1-copia retrotransposon family from cereal rye (Secale cereale L.). Genome / National Research Council Canada = Genome / Conseil national de recherches Canada 44 (2):266-274

Fu S, Chen L, Wang Y, Li M, Yang Z, Qiu L, Yan B, Ren Z, Tang Z (2015) Oligonucleotide probes for ND-FISH analysis to identify rye and wheat chromosomes. Scientific Reports 5 (1):10552

Gehrke F, Ruiz-Duarte P, Schindele A, Wolf S, Puchta H (2023) An inducible CRISPR-Kill system for temporally controlled cell type-specific cell ablation in Arabidopsis thaliana. New Phytol 239 (5):2041-2052. doi:10.1111/nph.19102

Guo J, Lei Y, Zhang H, Song D, Liu X, Cao Z, Chu C, Zhuang L, Qi Z (2019) Frequent variations in tandem repeats pSc200 and pSc119. 2 cause rapid chromosome evolution of open-pollinated rye. Molecular Breeding 39:1-13

Hashizume R, Wakita S, Sawada H, Takebayashi SI, Kitabatake Y, Miyagawa Y, Hirokawa YS, Imai H, Kurahashi H (2025) Trisomic rescue via allele-specific multiple chromosome cleavage using CRISPR-Cas9 in trisomy 21 cells. PNAS Nexus 4 (2):pgaf022. doi:10.1093/pnasnexus/pgaf022

Huang X, Zhu M, Zhuang L, Zhang S, Wang J, Chen X, Wang D, Chen J, Bao Y, Guo J (2018) Structural chromosome rearrangements and polymorphisms identified in Chinese wheat cultivars by high-resolution multiplex oligonucleotide FISH. Theoretical and Applied Genetics 131:1967-1986

Ishii T, Schubert V, Khosravi S, Dreissig S, Metje-Sprink J, Sprink T, Fuchs J, Meister A, Houben A (2019) RNA-guided endonuclease - in situ labelling (RGEN-ISL): a fast CRISPR/Cas9-based method to label genomic sequences in various species. New Phytol 222 (3):1652-1661. doi:10.1111/nph.15720

Kapusi E, Ma L, Teo CH, Hensel G, Himmelbach A, Schubert I, Mette MF, Kumlehn J, Houben A (2012) Telomere-mediated truncation of barley chromosomes. Chromosoma 121:181-190

Langmead B, Salzberg SL (2012) Fast gapped-read alignment with Bowtie 2. Nature methods 9 (4):357-359

Li H, Handsaker B, Wysoker A, Fennell T, Ruan J, Homer N, Marth G, Abecasis G, Durbin R, Subgroup GPDP (2009) The sequence alignment/map format and SAMtools. bioinformatics 25 (16):2078-2079

Lopez-Delisle L, Rabbani L, Wolff J, Bhardwaj V, Backofen R, Grüning B, Ramírez F, Manke T (2021) pyGenomeTracks: reproducible plots for multivariate genomic datasets. Bioinformatics 37 (3):422-423

Lukaszewski AJ (1997) Construction of midget chromosomes in wheat. Genome 40 (4):566-569

Ma S, Wang M, Wu J, Guo W, Chen Y, Li G, Wang Y, Shi W, Xia G, Fu D (2021) WheatOmics: A platform combining multiple omics data to accelerate functional genomics studies in wheat. Molecular Plant 14 (12):1965-1968

Nelson AD, Lamb JC, Kobrossly PS, Shippen DE (2011) Parameters affecting telomere-mediated chromosomal truncation in Arabidopsis. The Plant Cell 23 (6):2263-2272

Potlapalli BP, Dassau F, Fuchs J, Sushmoy DR, Houben A (2025) CRISPR-CISH: an in situ chromogenic DNA repeat detection system for research and life science education. Chromosome Res 33 (1):7. doi:10.1007/s10577-025-09767-1

Potlapalli BP, Fuchs J, Rutten T, Meister A, Houben A (2024) The potential of ALFA-tag and tyramide-based fluorescence signal amplification to expand the CRISPR-based DNA imaging toolkit. J Exp Bot 75 (20):6244-6257. doi:10.1093/jxb/erae341

Potlapalli BP, Schubert V, Metje-Sprink J, Liehr T, Houben A (2020) Application of Tris-HCl allows the specific labeling of regularly prepared chromosomes by CRISPR-FISH. Cytogenet Genome Res 160 (3):156-165. doi:10.1159/000506720

Puchta H, Houben A (2024) Plant chromosome engineering - past, present and future. New Phytol 241 (2):541-552. doi:10.1111/nph.19414

Riley R, Chapman V (1958) Genetic control of the cytologically diploid behaviour of hexaploid wheat.

Samach A, Mafessoni F, Gross O, Melamed-Bessudo C, Filler-Hayut S, Dahan-Meir T, Amsellem Z, Pawlowski WP, Levy AA (2023) CRISPR/Cas9-induced DNA breaks trigger crossover, chromosomal loss, and chromothripsis-like rearrangements. Plant Cell. doi:10.1093/plcell/koad209

Sandery MJ, Forster JW, Blunden R, Jones RN (1990a) Identification of a family of repeated sequences on the rye B-chromosome. Genome / National Research Council Canada = Genome / Conseil national de recherches Canada 33 (6):908-913

Sandery MJ, Forster JW, Blunden R, Jones RN (1990b) Identification of a family of repeated sequences on the rye B chromosome. Genome / National Research Council Canada = Genome / Conseil national de recherches Canada 33 (6):908-913

Schindele A, Gehrke F, Schmidt C, Rohrig S, Dorn A, Puchta H (2022) Using CRISPR-Kill for organ specific cell elimination by cleavage of tandem repeats. Nat Commun 13 (1):1502. doi:10.1038/s41467-022-29130-w

Schindele P, Wolter F, Puchta H (2020) CRISPR guide RNA design guidelines for efficient genome editing. RNA Tagging: methods and protocols:331-342

Schubert I, Oud JL (1997) There is an upper limit of chromosome size for normal development of an organism. Cell 88 (4):515-520

Schubert I, Rieger R (1985) A new mechanism for altering chromosome number during karyotype evolution. Theor Appl Genet 70 (2):213-221. doi:10.1007/BF00275324

Sears E (1956) The transfer of leaf-rust resistance from Aegilops umbellulata to wheat.

Sears E, Okamoto M (1958) Intergenomic chromosome relationships in hexaploid wheat.

Sears ER (1954) The aneuploids of common wheat. University of Missouri, College of Agriculture, Agricultural Experiment Station,

Shi F, Endo TR (1997) Production of wheat-barley disomic addition lines possessing an Aegilops cylindrica gametocidal chromosome. Genes & Genetic Systems 72 (4):243-248

Tamilselvan-Nattar-Amutha S, Dreissig S, Kumlehn J, Heckmann S (2023) Barley stripe mosaic virus-mediated somatic and heritable gene editing in barley (Hordeum vulgare L.). Frontiers in Plant Science 14:1201446

Teo CH, Ma L, Kapusi E, Hensel G, Kumlehn J, Schubert I, Houben A, Mette MF (2011) Induction of telomere‐mediated chromosomal truncation and stability of truncated chromosomes in Arabidopsis thaliana. The Plant Journal 68 (1):28-39

Tomita M, Shinohara K, Morimoto M (2008) Revolver is a new class of transposon-like gene composing the Triticeae genome. DNA research 15 (1):49-62

Vershinin AV, Schwarzacher T, Heslop-Harrison JS (1995) The large-scale genomic organization of repetitive DNA families at the telomeres of rye chromosomes. The Plant Cell 7 (11):1823-1833

Wang W, Yu Z, He F, Bai G, Trick HN, Akhunova A, Akhunov E (2022) Multiplexed promoter and gene editing in wheat using a virus‐based guide RNA delivery system. Plant Biotechnology Journal 20 (12):2332-2341

Weiss T, Crisp PA, Rai KM, Song M, Springer NM, Zhang F (2022) Epigenetic features drastically impact CRISPR-Cas9 efficacy in plants. Plant Physiol 190 (2):1153-1164. doi:10.1093/plphys/kiac285

Xu C, Cheng Z, Yu W (2012) Construction of rice mini‐chromosomes by telomere‐mediated chromosomal truncation. The Plant Journal 70 (6):1070-1079

Yan X, Li C, Yang J, Wang L, Jiang C, Wei W (2017) Induction of telomere‐mediated chromosomal truncation and behavior of truncated chromosomes in Brassica napus. The Plant Journal 91 (4):700-713

Yu W, Han F, Gao Z, Vega JM, Birchler JA (2007) Construction and behavior of engineered minichromosomes in maize. Proceedings of the National Academy of Sciences 104 (21):8924-8929

Yu W, Lamb JC, Han F, Birchler JA (2006) Telomere-mediated chromosomal truncation in maize. Proceedings of the National Academy of Sciences 103 (46):17331-17336

Yuan J, Shi Q, Guo X, Liu Y, Su H, Guo X, Lv Z, Han F (2017) Site-specific transfer of chromosomal segments and genes in wheat engineered chromosomes. Journal of Genetics and Genomics 44 (11):531-539

Zhao Y, Huang Z, Zhou X, Teng W, Liu Z, Wang W, Tang S, Liu Y, Liu J, Wang W (2025) Precise deletion, replacement and inversion of large DNA fragments in plants using dual prime editing. Nature Plants:1-15

Zuo E, Huo X, Yao X, Hu X, Sun Y, Yin J, He B, Wang X, Shi L, Ping J, Wei Y, Ying W, Wei W, Liu W, Tang C, Li Y, Hu J, Yang H (2017) CRISPR/Cas9-mediated targeted chromosome elimination. Genome Biol 18 (1):224. doi:10.1186/s13059-017-1354-4
